# Reductive Photocycloreversion of Cyclobutane Dimers Triggered by Guanines

**DOI:** 10.1021/acs.joc.3c00930

**Published:** 2023-07-12

**Authors:** Gemma M. Rodríguez-Muñiz, Ana B. Fraga-Timiraos, Miriam Navarrete-Miguel, Ana Borrego-Sánchez, Daniel Roca-Sanjuán, Miguel A. Miranda, Virginie Lhiaubet-Vallet

**Affiliations:** † Instituto Universitario Mixto de Tecnología Química (UPV-CSIC), Universitat Politècnica de València, Consejo Superior de Investigaciones Científicas, 46022 Valencia, Spain; ‡ Instituto de Ciencia Molecular, Universitat de València, P.O.Box 22085, 46071 València, Spain

## Abstract

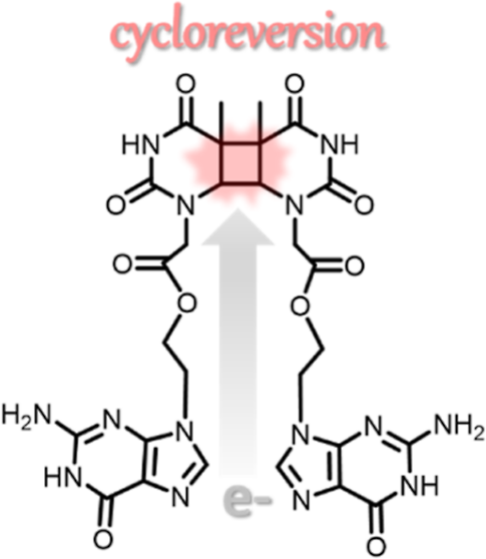

The quest for simple systems achieving the photoreductive splitting of four-membered ring compounds is a matter of interest not only in organic chemistry but also in biochemistry to mimic the activity of DNA photorepair enzymes. In this context, 8-oxoguanine, the main oxidatively generated lesion of guanine, has been shown to act as an intrinsic photoreductant by transferring an electron to bipyrimidine lesions and provoking their cycloreversion. But, in spite of appropriate photoredox properties, the capacity of guanine to repair cyclobutane pyrimidine dimer is not clearly established. Here, dyads containing the cyclobutane thymine dimer and guanine or 8-oxoguanine are synthesized, and their photoreactivities are compared. In both cases, the splitting of the ring takes place, leading to the formation of thymine, with a quantum yield 3.5 times lower than that for the guanine derivative. This result is in agreement with the more favored thermodynamics determined for the oxidized lesion. In addition, quantum chemistry calculations and molecular dynamics simulations are carried out to rationalize the crucial aspects of the overall cyclobutane thymine dimer photoreductive repair triggered by the nucleobase and its main lesion.

## Introduction

Splitting of four-membered ring compounds is an important process in organic chemistry for synthesizing molecules of different levels of complexity.^1−4^ Nature has also elegantly exploited this reaction to achieve efficient DNA repair of bipyrimidine lesions.^5,6^ The operating mechanism involves the action of enzymes called photolyases, which operate through a photoinduced electron transfer (eT) from a catalytic flavin–adenosine cofactor to the dimeric lesion. Over the years, the quest for simpler systems or models mimicking this activity has been a matter of interest.^4,7−12^ In this context, the photoredox properties of guanine (G) and its derivatives have fueled interest on whether these nucleobases can themselves photoinduce DNA repair. The first plausible hypothesis was proposed for photoreactivation of the main UV-induced lesion, the cyclobutane pyrimidine dimer (CPD), by deoxyribozymes.^5^ This self-healing process was assumed to follow a mechanism analogous to that used by photolyases, with the high-order G quadruplex structure acting as the light harvesting antenna.^5^ The excited G then donates an electron to a nearby CPD, catalyzing its photoreversal to the original pyrimidine bases.^13^ Experimental results appeared, at first sight, to support this mechanism. They included the strong inhibitory effect of G on UVB- and UVC-induced CPD formation, as well as the evidence that the dimer quantum yield is strongly dependent on the oxidation potential of the flanking bases, which is lower for purines than for pyrimidines.^14,15^ However, controversy appeared raising the importance of base conformation and/or excited-state delocalization.^16−18^ In this respect, experiments performed with 3- or 4-mers concluded on the inability of G to repair the CPD,^19^ but instead an exciplex-mediated eT was proposed as the photoactive species when -GA- tracks are present at the 5′-side of the dimer.^20^ Computational chemistry also proposed different hypotheses for the self-photorepair of the 5′-GA-CPD sequence. Most works supported that the excitation remains basically local^21,22^ with a sequential eT process involving multiple changes of the orbital character of S_1_ after local excitation of one of the purine base,^21^ whereas another one supported the exciplex scenario.^23^ In addition, the inability of G to repair CPD in oligonucleotides does not seem to be due to the low “driving force” of the electron injection process, because photoreductants with lower reduction potential in the excited state, such as pyrene (*E*_D_* = −1.8 V vs NHE)^24^ tethered to the oligonucleotide sequence, have been shown to inject electron into the helix toward pyrimidines with free-energy changes, Δ*G*, as low as −0.6 eV for the eT step. A similar Δ*G* value can be determined for the CPD repair in hairpins photoinduced by flavin inserted as a cap.^25^

A clear example of CPD photorepair triggered by a DNA component corresponds to the photoinduced eT from 8-oxo-7,8-dihydroguanine (OG), which is a common oxidatively generated lesion in DNA.^26−28^ Actually, the greatly lower redox potential of 0.74 V vs NHE for OG represents ≈0.6 V decrease with respect to G potential,^29^ leading to a more favorable eT to CPD. In this context, this oxidized photoproduct of guanine has been suggested as an early redox coenzyme in RNA-based catalysis, acting prior to the evolution of more sophisticated cofactors such as flavin adenine dinucleotide and repairing the photodamages through a photolyase-like activity.^28^ As in the case of G, the yield of photoreversal is highly dependent on the OG location with a more efficient photorepair when OG is stacked on the 5′ side of CPD than when located on the 3′ side. Moreover, a three- to fourfold more rapid splitting occurs if OG and CPD are placed in the same strand, compared to having them in complementary strands.^26^ The photorepair was also evidenced intermolecularly but with a very low bimolecular rate for the reaction.^27^

This background revealed how difficult is the study of long-distance charge transport through DNA because the results depend on the redox potential of the donor and the acceptor, on the distance and sequence in between, but also on the structure of the DNA since distortions may perturb the orbital overlap pattern. Hence, the capability of G as a single entity to act as a photoreductant for CPDs has not been proven yet. Therefore, in this work we design two synthetic systems containing G or OG as electron donating moiety and a CPD as acceptor. The efficiency of the photorepair was investigated and compared for these two models by a combined experimental and theoretical approach.

## Results and Discussion

### Synthesis

**Scheme 1:**
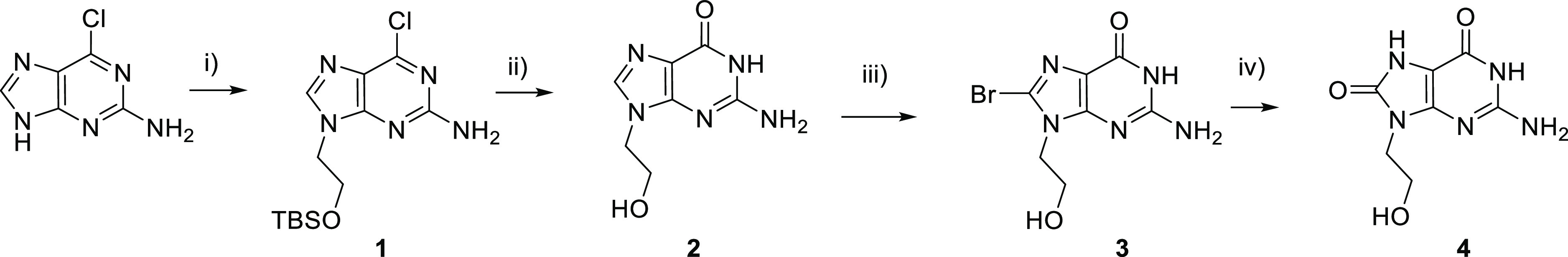
Synthetic Strategy to Prepare **2** and **4** Reagents and conditions: (i) 2-bromoethoxy-*tert*-butyldimethylsilane, NaH, DMF, rt, 24 h; (ii) 2 M HCl, 100 °C, 2 h; (iii) NBS, MeCN/H_2_O, rt, 30 min; (iv) AcONa, AcOH, 130 °C, 7 h.

**Scheme 2:**
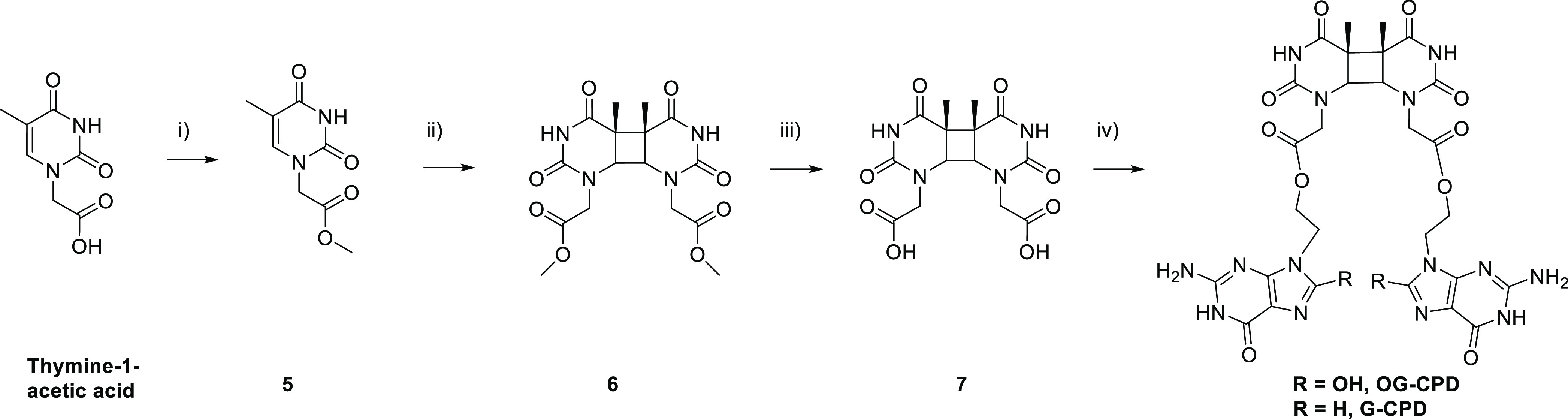
Synthetic Strategy to Prepare **G-CPD** and **OG-CPD** Reagents and conditions: (i) H_2_SO_4_, MeOH, 100 °C, 24 h; (ii) acetone/MeCN, hυ, λ > 290 nm, 72 h; (iii) 5 M HCl, 100 °C, 30 min; (iv) **2** or **4**, EDC, TBTU, DMAP, DMF, rt, 24 h.

The two models containing OG or G nucleobases covalently attached to a cyclobutane thymine dimer were synthesized (**OG-CPD** and **G-CPD**) following the methodology described in Schemes 1 and 2. On the one hand, the hydroxyethyl derivatives **2** and **4** were obtained after two and four steps, respectively (Scheme 1). First, the linker was easily introduced at N9 of 2-amino-6-chloropurine through alkylation with 2-bromoethoxy-*tert*-butyldimethylsilane to afford **1** with a high selectivity, this step was followed by hydrolysis to give the deprotected compound **2**. Subsequent bromination at the C8 position of **2** and hydrolysis with sodium acetate in acetic acid led to compound **4**.^30^

**Scheme 3:**
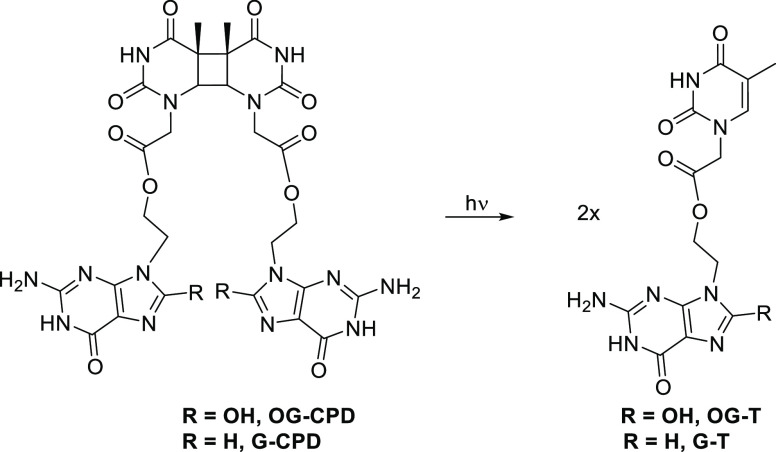
Cleavage Reaction of **OG-CPD** and **G-CPD** to Form **OG–T** or **G–T**, Respectively

On the other hand, the preparation of the *cis-syn* thymine dimer was performed in four steps from thymine-1-acetic acid as depicted in Scheme 2.^12,31^ First, the methyl ester **5** was prepared by Fischer esterification; then it was irradiated in a Pyrex vessel (λ > 290 nm) with a medium pressure mercury lamp (125 W) using acetone as photosensitizer to give a mixture of four isomers. The *cis-syn* thymine dimer **6** was separated by flash chromatography and its structural assignment was confirmed by ^1^H and ^13^C NMR by comparison with the spectra already described in the literature.^12,31^ Hydrolysis yielded the acetic acid derivative **7**, and subsequent esterification with **2** and **4** gave the corresponding **G-CPD** and **OG-CPD**, respectively. The repaired systems (**OG–T** and **G–T**, Scheme 3) were obtained with conditions similar to those described in Scheme 2, step (iv), but using thymine-1-acetic acid and **2** or **4** as starting material.

### Steady-State Photolysis

**Figure 1:**
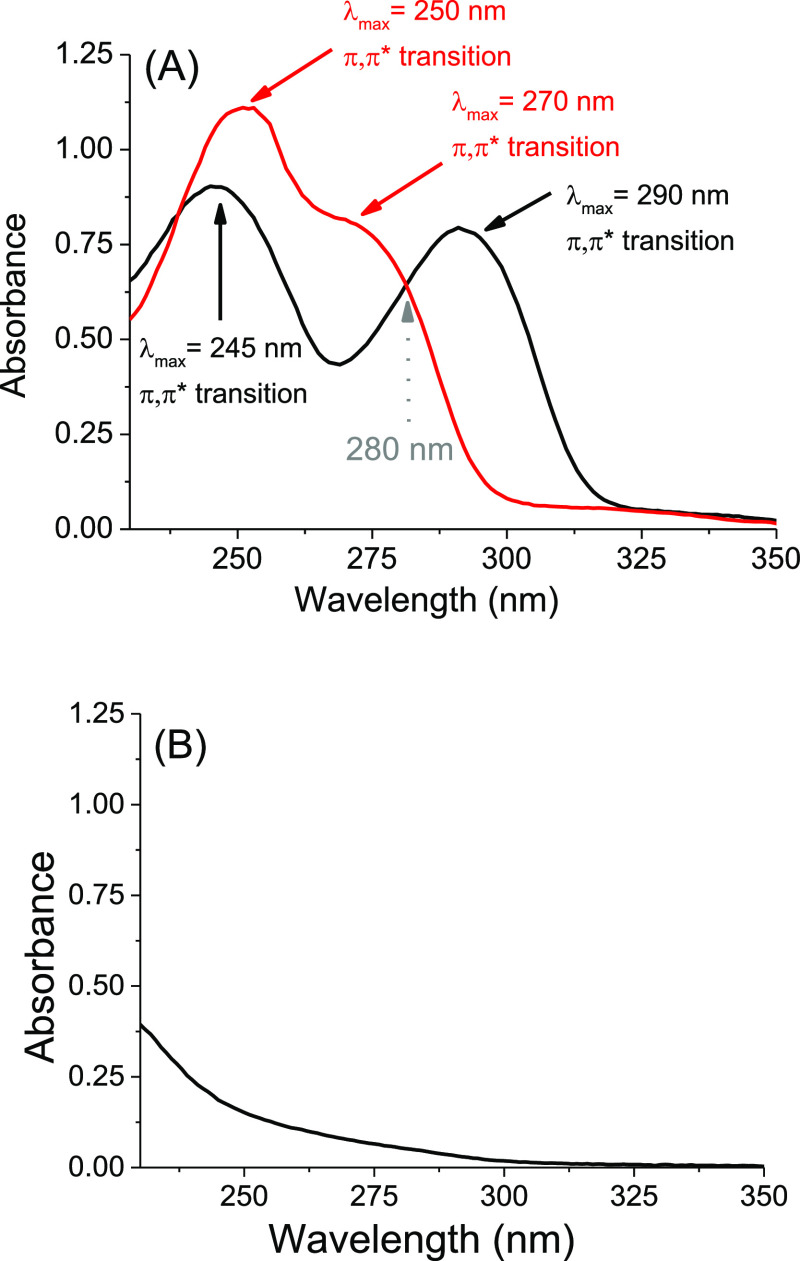
UV absorption spectra of (A) **OG-CPD** (0.1 mM, black) and **G-CPD** (0.1 mM, red) in PBS at pH 7.4; (B) **6** (0.1 mM) in MeCN. Absorption bands were assigned from refs (32) and (33).

The UV absorption spectra of **G-CPD** and **OG-CPD** are shown in Figure 1A. For **OG-CPD**, the characteristic band of OG chromophore with a maximum at ca. 290 nm is observed, this absorption is red shifted by respect to that of the guanine derivative **G-CPD**. In order to compare the splitting of the four-membered ring models, steady-state photolysis was performed using a monochromatic excitation at 280 nm. At this wavelength the two compounds are isoabsorptive in phosphate-buffered saline (PBS) solution at pH 7.4. Therefore, the splitting is achieved, for the same irradiation time, by the same number of absorbed photons for the two systems. In addition, at this wavelength and under the conditions of concentration used, the absorption of the CPD is negligible (Figure 1B), ensuring that the photons are mainly absorbed by the OG and G chromophores.

**Figure 2:**
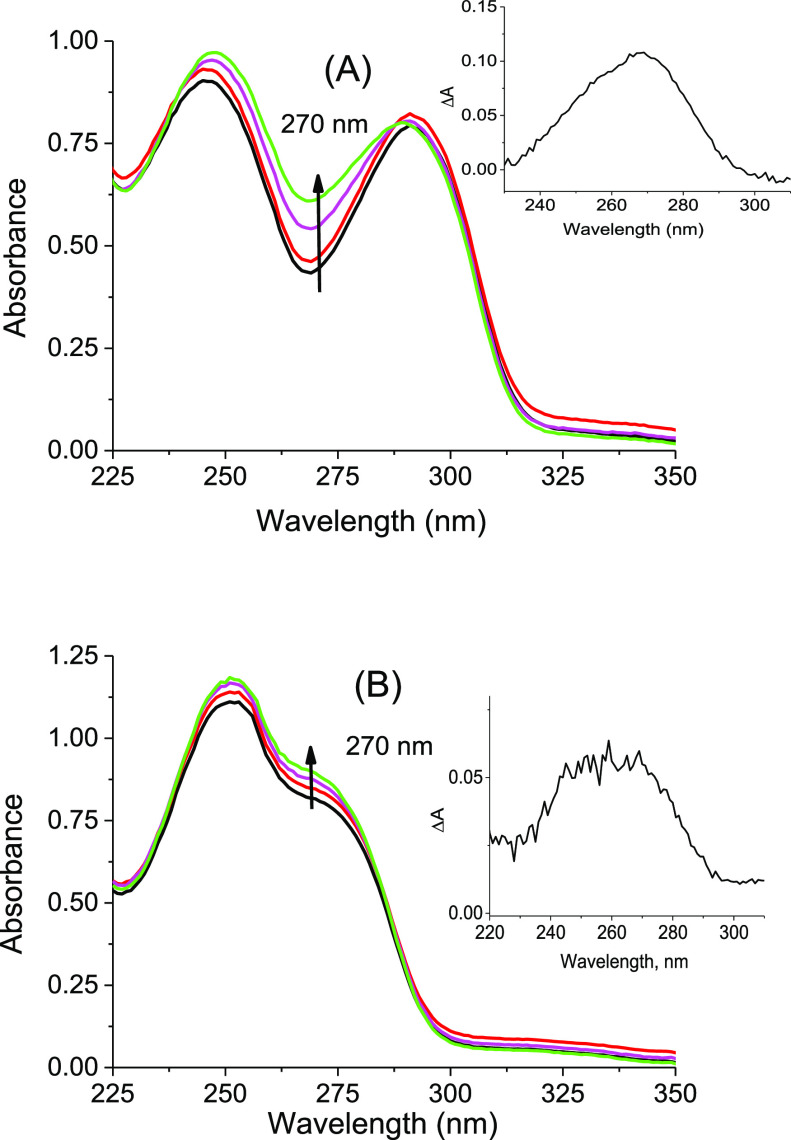
UV absorption spectra of (A) **OG-CPD** (0.1 mM) and (B) **G-CPD** (0.1 mM) in PBS at pH 7.4 obtained after 0 (dark line), 20 (red line), 40 (pink line), and 60 min (green line) of irradiation at 280 nm. Inset: difference spectra.

First, the photoreactivity of the models **G-CPD** and **OG-CPD** under 280 nm light was followed by UV spectrophotometry. The absorption changes were monitored at 270 nm to get information on the generation of the thymine chromophore present in the purported photoproducts **G–T** and **OG–T** (Scheme 3). As shown in Figure 2, steady-state photolysis of an aqueous solution (0.1 mM in PBS, pH 7.4) of both **OG-CPD** or **G-CPD** results in an increase at 270 nm. However, the changes are more pronounced in the case of the OG-derived system, pointing toward a higher efficiency of the process when the oxidized guanine acts as photoreductant.

**Figure 3:**
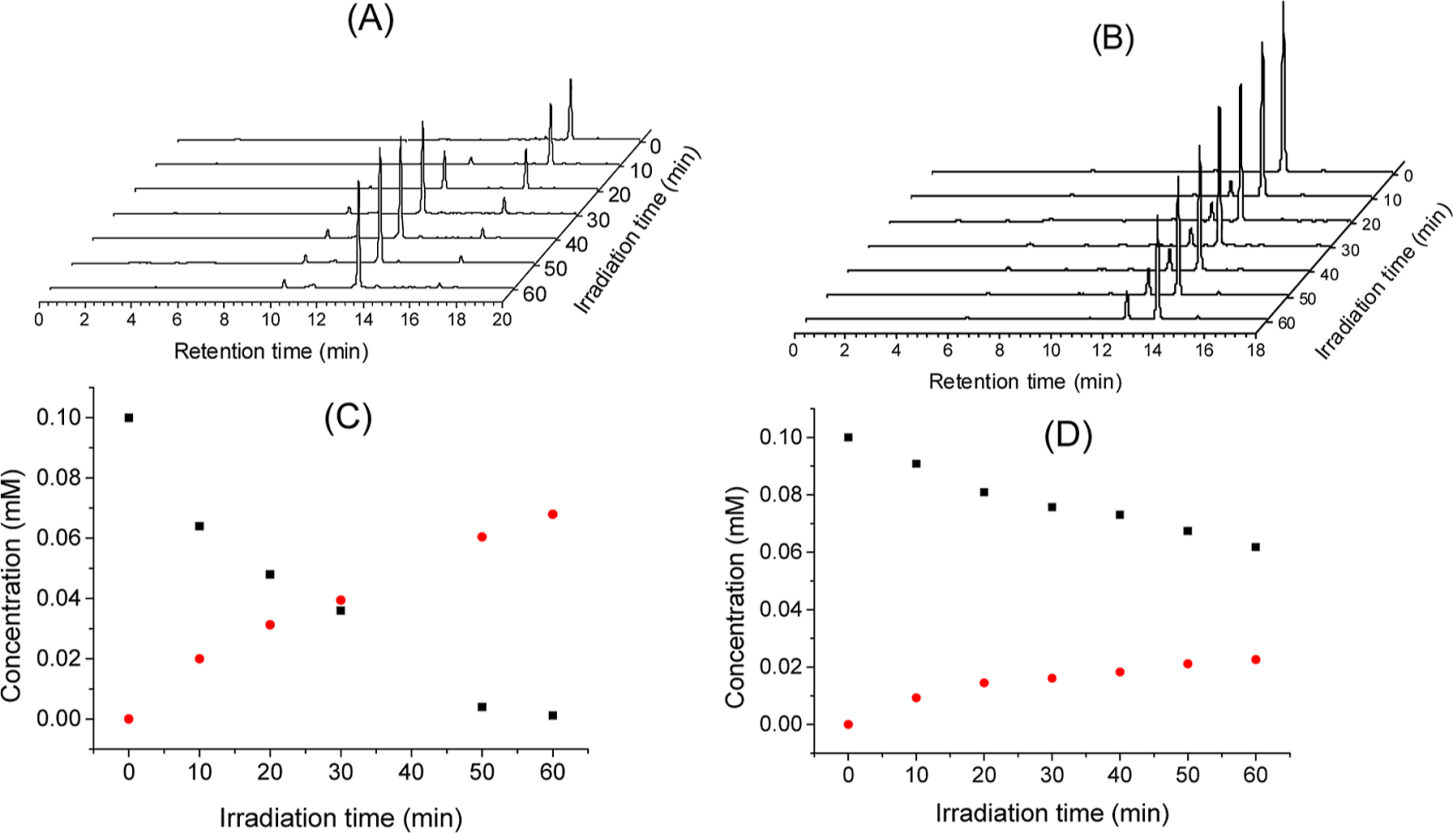
HPLC chromatograms obtained after 0, 10, 20, 30, 40, 50, and 60 min of irradiation of **OG-CPD** [0.1 mM, (A)] and **G-CPD** [0.1 mM, (B)] in PBS at pH 7.4 with 280 nm light. Kinetics of CPD splitting (black squares) and thymine formation (red dots) for **OG-CPD** (C) and **G-CPD** (D) systems.

In order to ensure that the photocleavage resulted in the expected photoproduct, the course of the reaction was monitored by HPLC at different irradiation times (Figure 3). In both **OG-CPD** and **G-CPD** cases, the initial CPD-derived systems, eluting at 16.8 min for **OG-CPD** and 13.8 min for **G-CPD**, are consumed over time to give rise to a new peak with a retention time of 13.2 and 12.6 min, respectively. This photoproduct was identified as **OG–T** for the **OG-CPD** photolysis and **G–T** for **G-CPD** by comparison with the synthetized samples (see the Experimental Section).

Quantification was done using authentic samples of the four compounds. After 30 min, almost 50% of the initial **OG-CPD** had reacted, while after 1 h, it was almost totally photolyzed (Figure 3A,C). Interestingly, under the same experimental conditions, only 20% of **G-CPD** had been consumed within the first 20 min and 40% after 2 h of irradiation (Figure 3B,D), which clearly demonstrates the higher photorepair efficiency of the OG moiety compared to G. To further compare both photoreactions, consumption quantum yields were determined for the CPD systems by means of an established procedure using as standard the photolysis of uridine in aerated water.^34^ The value obtained when OG acts as a photoreductant, **OG-CPD**, ϕ_OG-CPD_ = 7 × 10^–3^, is more than three times higher than when G is the electron donor, ϕ_G-CPD_ = 2 × 10^–3^.

These values are in the same order of magnitude as those of previous studies reporting a cleavage quantum yield much less than unity for a thymine dimer covalently tethered to an electron-donating chromophore such as phenothiazine, indole, or flavin.^10−12^

In this context, fluorescence experiments can inform on the photoinduced eT mechanism from the singlet excited state of the photoreductant. However, given the ultralow fluorescence quantum yields of G and OG (Φ_F_ of ca. 2.3 and 1.3 × 10^–4^, respectively),^32^ steady-state experiments run at room temperature did not show any significant signal of the dyad emission. In addition, OG and G exhibit an ultrafast fluorescence decay with an average lifetime ⟨τ⟩ of ca. 0.7 and 0.33 ps, respectively, the main deactivation pathway taking place through radiationless transition to the ground state.^32^ The radiative rate constants (*k*_F_), obtained from eq 1, are of ca. 10^8^ M^–1^ for OG and G (Table 1).
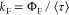
1

**Table 1:** Experimental Data of OG and G and Free-Energy Changes Calculated for the eT Process

	*E*_red_ (D/D^+•^) (V vs NHE)	*E*_red_ (D/D^+•^)* (V vs NHE)	*E*_S_* (eV)	Δ*G* (eV)	Φ_F_	*k*_F_ (s–^1^)
OG	0.74^a^	–3.16	3.90^b^	–1.20	1.3 × 10^–4^	1.9 × 10^8^
G	1.3^a^	–2.90	4.10^c^	–0.84	2.3 × 10^–4^	7.0 × 10^8^

a From ref (26).

b Determined from the intersection of fluorescence emission at 77 K and absorption spectra of **OG-CPD** (see Figure 4).

c Determined from the intersection of fluorescence emission at 77 K and absorption spectra of **G-CPD** (see Figure 4).

These values are of the same order of magnitude as those described for intramolecular eT (*k*_q_).^35−37^ Considering a *k*_q_ value of ca. 10^8^–10^9^ M^–1^, the changes in the fluorescence lifetime of the photoreductant (**G** or **OG**) can be evaluated from eq 2
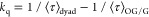
2where ⟨τ⟩_dyad_ is the average fluorescence lifetime of **OG-CPD** or **G-CPD**, and ⟨τ⟩_OG/G_ is the average fluorescence lifetime of **OG** or **G**. Therefore, due to the ultrafast kinetics of the photoreductant, shorter than 1 ps, the difference should be less than 0.5%, i.e., 10^–3^ ps, a scale that is far below the time resolution using upconversion fluorescence. This was confirmed by studying the **OG-CPD** model, for which the most efficient quenching is expected. The emission signal at 360 nm was monitored after 267 nm excitation (Figure S2) and compared to that of compound **4**, used as a reference to evaluate the quenching of the OG singlet excited state by the CPD. As reported for OG,^32^ kinetics cannot be fitted in a satisfactory way by a monoexponential function. A biexponential function with an additional small-amplitude component accounting for a long-lived residual fluorescence (representing less than 5% of the total signal) reproduces the data more adequately. Results of the fits are gathered in Table S1. The obtained average lifetimes ⟨τ⟩ of ca 0.42–0.45 ps were somewhat shorter than that of isolated OG.^32^ However, no significant difference was observed between the decays obtained for **OG-CPD** and compound **4** (Figure S2).

The efficiency and ability of photosensitizers to induce a reductive dimer splitting is related to their reduction potential in the excited-state, *E*_red_(D/D^+•^)*, which was determined from eq 3. Table 1 summarized the reduction potentials *E*_red_(D/D^+•^) obtained from the literature,^26^ and the experimental data of the singlet excited state energy, *E*_S_*. These values were calculated with the Planck–Einstein relation using λ_0–0_, the wavelength corresponding to the intersection between the normalized fluorescence emission at 77 K and the absorption signal of the dyads (Figure 4). Energies *E*_S_* of ≈3.90 and 4.10 eV were determined for **OG-CPD** and **G-CPD**, respectively.
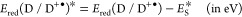
3

**Figure 4:**
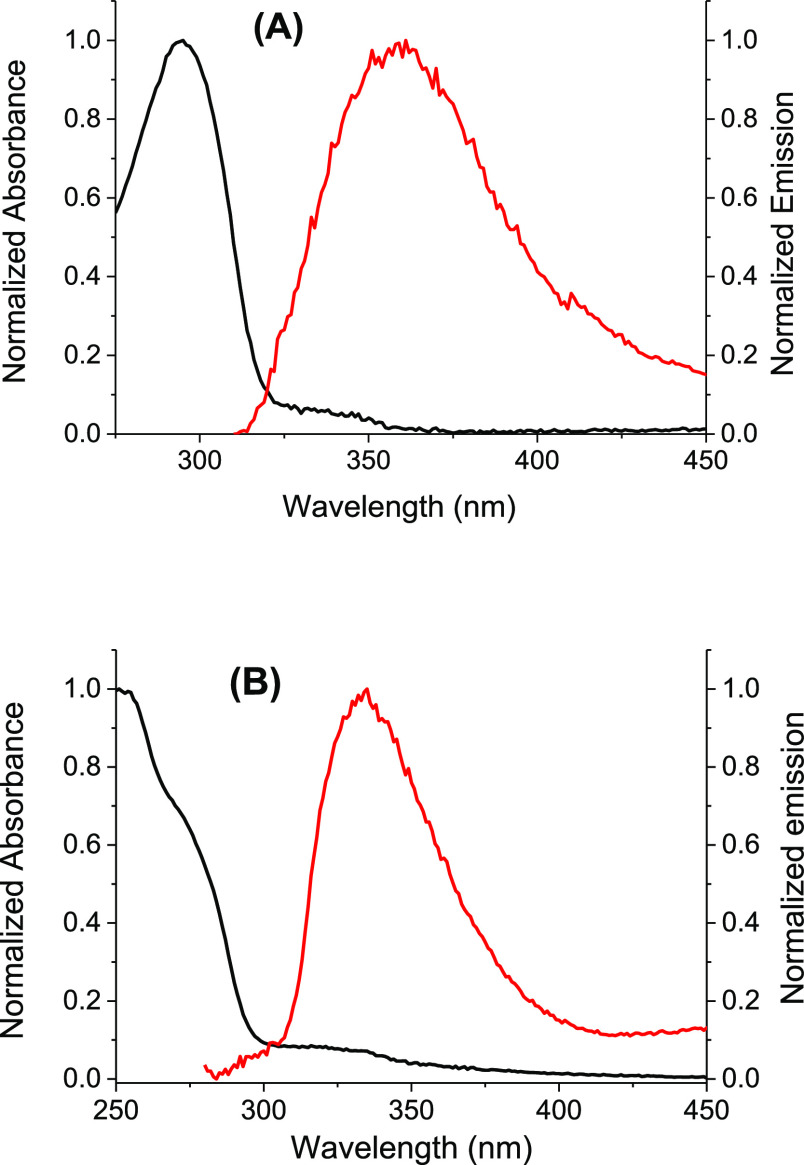
Normalized absorption (black line) and emission (red line) spectra of **OG-CPD** (A) and **G-CPD** (B) in EtOH glass at 77 K. λ_exc_ = 295 nm.

Interestingly, the OG has a more negative *E*_red_(D/D^+•^)* than G (Table 1), which is in line with its higher efficiency for cycloreversion. To know if the photoinduced eT from G and OG to CPD is an energetically favorable process, the associated Gibbs free energy (Δ*G*) was determined according to eq 4 and using the previously reported *E*_red_(A^–•^/A) of ca. −1.96 V vs NHE for CPD.^38^
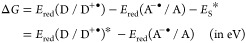
4

As shown in Table 1, favorable thermodynamics is anticipated for eT quenching for both compounds. The process is more favorable for OG, being more exergonic as *E*_red_(D/D^+•^)* becomes more negative.

### Quantum Chemistry Computations

Density functional theory (DFT) at the B3LYP/6-311++G(2df,p)//B3LYP/6-31+G(d) level was used to further rationalize the molecular basis of the photoreduction step taking place between the excited G* or OG* and the CPD, giving rise to G^•+^ or OG^•+^ and CPD^•–^. For convenience, this process can be considered as the combination of three steps: the generation of the excited state in G and OG, the extraction of an electron from these systems, and the injection of the electron into the CPD. The energetics can be approximated theoretically by eq 5
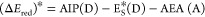
5where AIP(D) is the adiabatic ionization potential of G or OG, *E*_S_*(D) is the absorption band origin of G or OG, and AEA(A) is the adiabatic electron affinity of CPD.

Regarding the first step (generation of the singlet excited state), we must point out that no significant minima can be found either for G or for OG,^32,39^ as the molecule decays toward a conical intersection (CI). This correlates with the very weak fluorescence observed in the experiments and points to a strong competitive process, i.e., internal conversion, leading back to the ground state. It shall significantly contribute to the decrease in the efficiency of the eT step. Regarding the reduction properties of G and OG, both experimental and theoretical magnitudes, *E*_red_(D/D^+•^) and AIP, are related to each other and coincide, leading to a higher ability of OG with respect to G to donate an electron to the CPD (see the computed values in Table 2). On the other hand, the computed AEA of the CPD in the gas phase (0.88 eV) and in water solution (2.42 eV) show a large stabilization of the ionic state under the latter conditions in which the experiments are done. The energetics (Δ*E*_red_)* of this eT step from the singlet excited state of OG or G to the CPD were evaluated using eq 5, using the experimental value for *E*_S_* (Table 1) and the computed values for AIP and AEA (Table 2). A more negative (and therefore more favorable) value was obtained in the case of OG. Even though different approximations were used in the quantum chemistry computations, a qualitatively agreement is obtained with the experimental estimation of Δ*G* reported in Table 1. The exothermicity of the process was also supported by Anusiewicz et al. in a computational study in a duplex DNA.^40^

**Table 2:** Data (in eV and in kcal mol^–1^ within Parentheses) to Assess the Ability of OG and G to Photoinduce the CPD Ring Opening, Experimental Absorption Band Origin (*E*_S_*), Computed AIP, Calculated Energy Change (Δ*E*_red_)*, Related to the Photoreduction (OG*/G* + CPD → OG^•+^/G^•+^ + CPD^•–^), and Energy Barrier (Δ*E*_pc_^⧧^) for the Overall Process OG*/G* to the CPD Split Product (See the Text)^a^

	*E*_S_* (exp)	AIP(D)	(Δ*E*red)*	Δ*E*_pc_^⧧^
OG	3.90 (90.0)	5.48	–0.84 (−19.4)	–0.74 (−17.1)
G	4.10 (94.6)	5.78	–0.75 (−17.2)	–0.65 (−14.9)

a Calculations were performed in water solution.

Once the charged species G^•+^ or OG^•+^ and CPD^•–^ are produced, the cyclobutane ring split to produce the thymine monomers. This step of CPD^•–^ ring opening was studied theoretically by Durbeej and Eriksson also using DFT and the B3LYP functional.^41^ They found that the addition of an electron to the CPD directly induces the C_5_–C_5_′ cleavage, not finding any transition state (TS) for this process. After this, the C_6_–C_6_′ bond breaking goes through a TS, with an activation energy (Δ*E*^⧧^) of 2.3 kcal mol^–1^ and the split anionic product lies 2.4 kcal mol^–1^ below the CPD radical anion. A similar bond-breaking mechanism and an almost identical energy barrier was reported from a computational study on a duplex DNA (2.5 kcal mol^–1^).^42^ The exothermicity of the bond breakings upon electron attachment was also supported by Barbatti.^43^

Overall, the differential ability of OG and G from their singlet excited state OG* and G* to complete the cycloreversion of the CPD, Δ*E*_pc_^⧧^, can be approximated by using eq 6
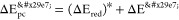
6

This magnitude considers and connects the excitation of G and OG, the eT process to CPD, and the kinetics of the cyclobutane ring opening (see more details on this approach to model the phenomenon in our previous work, Navarrete-Miguel et al.).^44^ Note that the release of energy occurring in the eT to generate the charged species G^•+^ or OG^•+^ and CPD^•–^, (Δ*E*_red_)*, is clearly higher as compared to the small energy barrier needed to break the ring in the CPD^•–^ system, Δ*E*^⧧^. The total energy balance, Δ*E*_pc_^⧧^, is more negative for OG, which should contribute to favoring the phenomenon in OG.

### Molecular Dynamics Simulations

Geometrical arrangement between the donor and acceptor moieties is also an important parameter for quenching by charge transfer. To this aim, molecular dynamics were performed to analyze the conformations accessible and more favorable for the **G-CPD** and **OG-CPD** in PBS.

**Figure 5:**
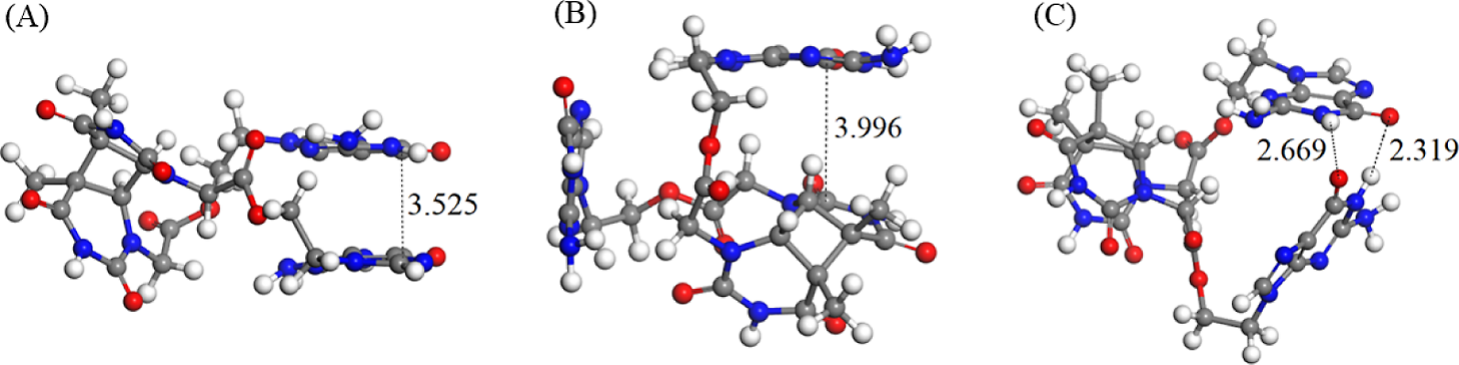
Stable conformations of **G-CPD** during the MD simulation. G–G π-stacking (A), G-T π-stacking (B), and G–G hydrogen bonding (C). Relevant atom distances (in Å) are highlighted (see numbering in Figure S3).

In **G-CPD**, the analysis of the structures visited along the simulations shows fast and frequent conformational changes. We identify three types of conformations present in this system that are relevant for the interpretation of the experimental observations: π-stacking between the two guanine monomers (G–G π-stacking, Figure 5A), π-stacking between a guanine and a thymine moiety of the CPD (G-T π-stacking, Figure 5B), and hydrogen bonding between the guanine monomers (G–G hydrogen bonding, Figure 5C). Such conformations appear in 7.4, 7.0, and 2.5%, respectively, of the explored conformational space throughout the molecular dynamics simulations. The G–G π-stacking conformation will stabilize the excited-state minimum by excimer interaction, thereby decreasing the competitive process of internal conversion. However, the photoreductant (G) is here far from CPD, which should make the eT difficult. In this aspect, the G-T π-stacking conformation shall contribute positively by helping to donate the electron by π-stacking.^45^ The last type of conformation (G–G hydrogen bonding) is expected to decrease the efficiency of eT since the CPD is far from the G monomers and the proton transfer process might happen decreasing the excited-state lifetime of the photoreductant agents.

**Figure 6:**
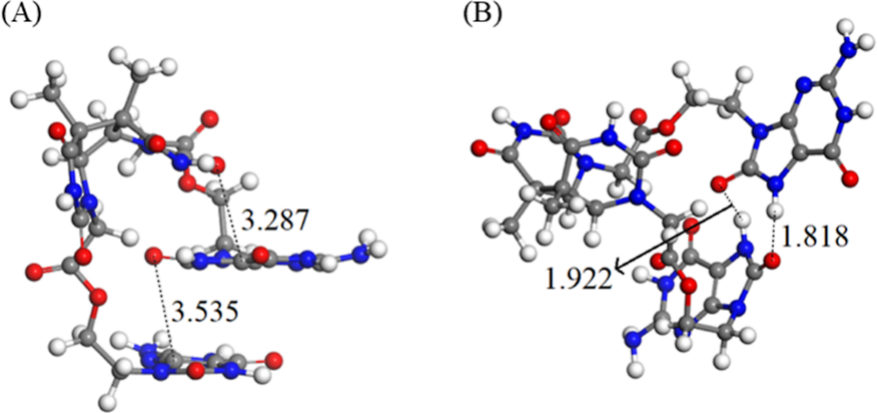
Stable conformations of **OG-CPD** during the MD simulation. OG–OG–T π-stacking (A), and OG–OG hydrogen bonding (B). Relevant atom distances (in Å) are highlighted (see numbering in Figure S4).

In the case of **OG-CPD**, the molecular dynamics simulations showed much more stable conformations over time than that observed in the previous system (**G-CPD**). Two major conformations are mainly present: π-stacking trimer between the oxoguanine molecules and the thymine moiety of the CPD (OG–OG–T π-stacking, Figure 6A) and hydrogen bonding conformation between the oxoguanines (OG–OG hydrogen bonding, Figure 6B). They appear in 58.2 and 22.4% of the conformations in the molecular dynamics simulations. The former structure, not observed in **G-CPD**, is expected to play a more relevant role in the eT process by decreasing the competition of internal conversion due to excimer interaction of the oxoguanines and facilitating electron donation to CPD via the π-stacking arrangement between the oxoguanine in the middle and the thymine part of the CPD. Regarding the OG–OG hydrogen bonding conformation, it has been observed to decrease the overall efficiency, as reported for **G-CPD**.

Among the two model systems, **OG-CPD** presented a more favorable geometric arrangement of the two interacting moieties (Figure 6A), where the stacking between the donor and the acceptor should enhance the eT. This conformation, present with a 58.2% frequency, should boost the eT with respect to that occurring in the **G-CPD** dyad, where the favorable geometric arrangement of the two interacting moieties (Figure 5B) exists with a low frequency of 7.0%.

However, all photoinduced eT-initiated chemical reactions compete with an exothermic back-electron-transfer (beT) process, and quantum yields approaching unity are only reached when the rate of the chemical process is faster relative to that of the beT. In other words, after excitation of the photoreductant and eT, the radical pair (G^•+^-CPD^•–^ or OG^•+^-CPD^•–^) can undergo two processes: CPD splitting, leading to repair, or beT, which corresponds to an unproductive reaction. Indeed, the proximity of the G (or OG) and CPD moieties acts as a double-edged sword, favoring both eT and beT. Thus, in our systems and as described for other synthetic models,^10−12^ the low quantum yields of CPD repair can be mainly associated with an efficient beT, which prevails on the splitting process, but also with rapid internal conversion of the excited chromophore.

In this context, Nature has endowed the CPD photolyase enzymes with the optimal conditions for effective photorepair, achieving reversal yields close to unity.^46^ This maximized efficiency is provided, on the one hand, by a rigid active site to avoid the ultrafast deactivation of the flavin cofactor, lengthening the singlet excited state lifetime in this manner. On another hand, a favorable redox environment results in the perfect interplay between a fast eT (236 ps) and a slow beT (2.4 ns).^46^

## Conclusions

Model systems of cyclobutane thymine dimers connected to guanine or 8-oxoguanine have been synthesized, and their photocycloreversion has been evaluated and compared. The photocleavage of the four-membered ring has been followed by UV spectrophotometry and HPLC. In agreement with the thermodynamics of the reaction, a more efficient cleavage is observed for OG than for G. The low quantum yields of photosplitting ϕ of ca. 10^–3^, are the result of a competition between the unproductive beT process and the repair channel corresponding to cleavage of the dimer radical anion. Theoretical calculations of the adiabatic ionization potential of OG/G and the adiabatic electron affinity of CPD corroborate these conclusions. In addition, molecular dynamics simulations show a stacking between the donor and acceptor moiety of OG-CPD, which should favor the charge transfer.

Altogether, these results show that independently of its presence in an oligonucleotide sequence and the possibility to form a GA exciplex, isolated G is also able to repair CPD by a photoinduced reductive reaction, although in a lower yield than that observed for OG.

## Experimental Section

### Chemicals

2-Amino-6-chloropurine, 2-bromoethoxy-*tert*-butyldimethylsilane, *N*-bromosuccinimide (NBS), sodium acetate (AcONa), glacial acetic acid (AcOH), *N*-(3-dimethylaminopropyl)-*N*′-ethylcarbodiimide (EDC), 4-dimethylaminopyridine (DMAP), 2-(1*H*-benzotriazole-1-yl)-1,1,3,3-tetramethylaminium tetrafluoroborate (TBTU), sodium hydride (NaH), and sulfuric acid were purchased from Sigma-Aldrich. Methanol (MeOH), dimethylformamide (DMF), and acetonitrile (MeCN) were purchased from Scharlab.

### Nuclear Magnetic Resonance

A Bruker 300 or 400 MHz spectrometer was used for the nuclear magnetic resonance (NMR) experiments. The signal of the solvent, dimethylsulfoxide, was used as a reference for the determination of the chemical shifts (δ) in ppm.

### High-Resolution Mass Spectrometry

The Waters ACQUITY XevoQToF Spectrometer (Waters Corp.) was connected to the UPLC system (ACQUITY UPLC system, Waters Corp.) via an electrospray ionization (ESI) interface. The separation was carried out on a Zorbax Eclipse Plus C18 (100 × 4.6 mm, 3.5 μm) column at a 0.4 mL/min flow rate and appropriate gradient of acidified (0.1% formic acid) acetonitrile and water. The ESI source was operated in the positive mode with a capillary voltage of 3.0 kV, and the temperatures of the source and desolvation set at 120 and 500 °C, respectively. All data collected in the centroid mode were acquired using MassLynx software (Waters Corp.). Leucine-enkephalin was used as the lock mass generating an [M + H]^+^ ion (*m*/*z* 556.2771) at a concentration of 500 pg/mL and a flow rate of 20 μL/min to ensure accuracy during the MS analysis.

### UV–Vis Absorption

UV absorption spectra were recorded on a Cary 50 spectrophotometer (Varian) using a quartz cuvette of 1 cm optical path and 3 mL capacity.

### Steady-State Photolysis

Samples were prepared in PBS solution at pH 7.4 at a concentration of 0.1 mM; their absorption at 280 nm, *A*_280_, was of ca. 0.75. All irradiations were carried out under oxygen-free conditions at room temperature using 3 mL quartz cuvettes of 1 cm optical path. Monochromatic irradiation (at λ_exc_ = 280 nm) was performed using a xenon lamp (75 W) coupled to a monochromator from Photon Technology International (PTI). The course of the photoreaction was followed by HPLC. The quantum yield of the photoreaction was established using uridine in water as an actinometer, ϕ_urd_ (280 nm) = 0.016.^34^ A solution with *A*_280_ = 0.8 was irradiated, and the course of the reaction was followed by UV–vis spectrometry.

### High-Performance Liquid Chromatography

The irradiated solutions were analyzed by HPLC using an Agilent 1100 instrument equipped with a UV detector; for all chromatograms, the detection wavelength was set at 270 nm. Samples were analyzed through a reverse phase a Mediterranea Sea C18 (4.6 mm i.d. × 25 cm length, 5 μm) column and using a linear gradient of 2:98 (MeCN/H_2_O) to 65:35 over 30 min at a 1 mL min^–1^ flow rate. The injection volume was 10 μL. The photodegradation and formation yields were determined from calibration curve using pure samples.

### Fluorescence Analysis

Emission spectra of **OG-CPD** and **G-CPD** were obtained on a FLS1000 spectrometer (Edinburgh Instruments) equipped with a 400 W xenon lamp, double grating Czerny-Turner monochromators with 2 × 325 mm focal length in excitation and detection, and a PMT-980 detector in a cooled housing which covers a range from 200 to 980 nm.

The samples were prepared in ethanol, with an absorbance of ca. 0.16 at the excitation wavelength (λ_exc_ = 295 nm), and cooled to 77 K using a cryostat (Optistat, Oxford instruments). Measurements were performed using low-temperature quartz cuvettes.

### Upconversion Fluorescence Analysis

These measurements were performed at SGIKER Laser Facility. Ultrashort laser pulses (35 fs, 800 nm) are produced in an oscillator–regenerative amplifier laser system (Coherent, Mantis-Legend). Pump pulses at 267 nm (0.6 μJ), generated by a third harmonic generation, are focused by a lens (*f* = 60 mm) on a rotatory cuvette of 0.4 mm path containing the solution under study. The sample emission is collected by an *f* = 60 mm lens and focused by a second one with *f* = 150 mm, on a 0.2 mm thick BBO crystal, where it interacts with the 800 nm probe beam to generate the sum frequency signal by type-I phase matching. The latter, coupled to a monochromator (CDP 220D), is detected by a photomultiplier (PMT), whose signal is integrated by a boxcar integrator (CDP 2021A). The relative delay between the excitation and probe pulses is controlled by a motorized translation stage with a maximum delay of ∼2 ns and a precision of 1.5 fs. The relative polarization of pump and probe beams is set to 54.7° to avoid contributions of rotational diffusion. Each measurement is the result of the average of 10 scans containing 1500 laser shots at each delay position. The instrumental response function is around 350 fs at the studied excitation and emission wavelengths. Concentrations of 21.6 mg of **4** and 31.0 mg of **OG-CPD** in 25 mL PBS (4% DMSO) were used.

### Synthesis

#### 2-Amino-6-chloro-9-(2-ethyloxy-*tert*-butyldimethylsilane)purine (**1**)

To an ice-cold solution of 2-amino-6-chloro purine (2.8 g, 16.5 mmol) in anhydrous DMF (25 mL) was added NaH (0.4 g, 17.9 mmol). After 30 min, 2-bromoethoxy-*tert*-butyldimethylsilane (4.3 g, 18.1 mmol) was added, and the mixture was stirred for 24 h at room temperature under nitrogen. Then, H_2_O (25 mL) was poured, and the mixture was filtrated and washed with cold water. The crude product was purified by column chromatography (silica gel/*n*-hexane/ethyl acetate, 2:3) to give **1** (2.4 g, 51%) as a white solid. ^1^H NMR (DMSO-*d*_6_, 300 MHz): δ 8.05 (s, 1H), 6.90 (s, 2H), 4.17 (t, 2H, *J* = 6 Hz), 3.90 (t, 2H, *J* = 6 Hz), 0.76 (s, 9H), −0.14 (s, 6H). ^13^C {^1^H }NMR (DMSO-*d*_6_, 75 MHz): δ 159.7 (C), 154.1 (C), 149.2 (C), 143.7 (CH), 123.3 (C), 60.5 (CH_2_), 45.4 (CH_2_), 25.5 (CH_3_), 17.7 (C), −5.80 (CH_3_). HRMS (ESI/Q-TOF): *m*/*z*: [M + H]^+^ calcd for C_13_H_23_N_5_OSiCl, 328.1344; found, 328.1360.

#### 9-(2-Hydroxyethyl)guanine (**2**)

A solution of **1** (2.4 g, 7.33 mmol) in 2 M HCl (22 mL) was heated under reflux using a heating mantle for 2 h. The solvent was neutralized and cooled with ice. The solid was filtered off and washed with cold H_2_O to give pure **2** (1.3 g, 87%). ^1^H NMR (DMSO-*d*_6_, 300 MHz): δ 10.56 (s, 1H), 7.64 (s, 1H), 6.44 (s, 2H), 5.00 (t, *J* = 6 Hz, 1H), 3.99 (t, *J* = 6 Hz, 2H), 3.69–3.63 (m, 2H). ^13^C {^1^H }NMR (DMSO-*d*_6_, 75 MHz): δ 156.8 (C), 153.4 (C), 151.1 (C), 137.9 (CH), 116.5 (C), 59.3 (CH_2_), 45.4 (CH_2_). HRMS (ESI/Q-TOF) *m*/*z*: [M + H]^+^ calcd for C_7_H_10_N_5_O_2_, 196.0825; found, 196.0834.

#### 8-Bromo-9-(2-hydroxyethyl)guanine (**3**)

In a round-bottom flask, **2** (1.3 g, 6.1 mmol) was dissolved in 62 mL MeCN and 15 mL H_2_O, and added with NBS (1.6 g, 9.1 mmol). The suspension was stirred for 30 min at room temperature and subsequently evaporated to dryness. The residual solid was taken up in 20 mL acetone and stirred for 30 min at room temperature. Subsequently, the mixture was filtered and washed with cold acetone and dried to yield **3** (0.85 g, 65%) as a beige solid. ^1^H NMR (DMSO-*d*_6_, 300 MHz): δ 10.67 (s, 1H), 6.58 (s, 2H), 5.0 (t, *J* = 6 Hz, 1H), 4.00 (t, *J* = 6 Hz, 2H), 3.70–3.64 (m, 2H). ^13^C {^1^H }NMR (DMSO-*d*_6_, 75 MHz): δ 155.5 (C), 153.7 (C), 152.5 (C), 121.3 (C), 116.8 (C), 58.6 (CH_2_), 46.0 (CH_2_). HRMS (ESI/Q-TOF): *m*/*z* [M + H]^+^ calcd for C_7_H_9_N_5_O_2_Br, 273.9952; found, 273.9940.

#### 9-(2-Hydroxyethyl)-8-oxoguanine (**4**)

Compound **3** (0.85 g, 3.1 mmol) was dissolved in a solution of sodium acetate (2.5 g, 31.14 mmol) in glacial acetic acid (113 mL). The reaction mixture was heated using a heating mantle at 130 °C for 7 h. Acetic acid was evaporated, and a residue was codistilled with water (3 × 20 mL). The residual solid was taken up in aqueous sodium hydroxide (0.1 M, 10 mL) and heated at reflux for 10 min. Then, it was acidified to pH 7 in an ice bath, and the resulting precipitate was filtered off and washed with water to give **4** (0.3 g, 46%) as a beige solid. ^1^H NMR (DMSO-*d*_6_, 300 MHz): δ 10.60 (s, 1H), 10.51 (s, 1H), 6.45 (s, 2H), 4.85 (t, *J* = 6 Hz, 1H), 3.68–3.63 (m, 2H), 3.60–3.57 (m, 2H). ^13^C {^1^H }NMR (DMSO-*d*_6_, 75 MHz): δ 153.4 (C), 152.4 (C), 150.9 (C), 148.1 (C), 98.2 (C), 58.2 (CH_2_), 41.6 (CH_2_). HRMS (ESI): *m*/*z* calcd for C_7_H_10_N_5_O_3_ [M + H]^+^, 212.0780; found, 212.0784.

#### Methyl 2-(Thymin-1-yl)acetate (**5**)

To a stirred solution of thymine-1-acetic acid (5 g, 27.1 mmol) in MeOH (200 mL), H_2_SO_4_ (1 mL) was added. The reaction was heated using a heating mantle to reflux overnight. The solvent was evaporated under pressure, diluted with H_2_O (100 mL), and cooled with ice. The solid was filtered off and washed well with cold H_2_O to give pure **5** (4.2 g, 78%) as a white solid. ^1^H NMR (DMSO-*d*_6_, 300 MHz): δ 11.42 (s, 1H), 7.52 (s, 1H), 4.50 (s, 2H), 3.70 (s, 3H), 1.78 (s, 3H). ^13^C {^1^H }NMR (DMSO-*d*_6_, 75 MHz): δ 168.7 (C), 164.3 (C), 150.9 (C), 141.5 (CH), 108.6 (C), 52.2 (CH_3_), 48.3 (CH_2_), 11.8 (CH_3_). HRMS(ESI): *m*/*z* calcd for C_8_H_11_N_2_O_4_ [M + H]^+^, 199.0717; found, 199.0719.

#### *cis*-*syn*-Cyclobutane Dimer of Methyl 2-(Thymin-1-yl)acetate (**6**)

The methyl ester **5** (4 g, 20.2 mmol) was dissolved in acetone/acetonitrile (500 mL, 1:4), and the solution was degassed for 30 min. The solution was irradiated for 72 h with a medium-pressure mercury lamp (125 W) in a Pyrex vessel. The reaction mixture was filtrated and evaporated to dryness in vacuo. The product was isolated by flash chromatography (silica gel/CHCl_3_/MeOH, 5:0.15) to give **6** as a white solid (0.3 g, 3.8%). ^1^H NMR (DMSO-*d*_6_, 300 MHz): δ 10.54 (s, 2H), 4.23 (d, *J* = 16.5 Hz, 2H), 3.98 (s, 2H), 3.88 (d, *J* = 16.5 Hz, 2H), 3.67 (s, 6H), 1.37 (s, 6H). ^13^C {^1^H }NMR (DMSO-*d*_6_, 75 MHz): δ 170.5 (2C), 169.2 (2C), 152.2 (2C), 59.4 (2CH), 51.9 (2CH_3_), 47.1 (2CH_2_), 46.2 (2C), 18.2 (2CH_3_). HRMS (ESI): *m*/*z* calcd for C_16_H_21_N_4_O_8_ [M + H]^+^, 397.1281; found, 397.1296.

#### *cis-syn*-Cyclobutane Dimer of Thymine-1-acetic Acid (**7**)

The diester **6** (200 mg, 0.5 mmol) was dissolved in 5 M hydrochloride (5 mL). The reaction mixture was refluxed using a heating mantle for 30 min, and then the reaction solution was concentrated in vacuo. The product was washed with Et_2_O and dried in vacuo to yield a white solid **7** (0.1 g, 62%). ^1^H NMR (DMSO-*d*_6_, 300 MHz): δ 12.83 (s, 2H), 10.47 (s, 2H), 4.19 (d, *J* = 15 Hz, 2H), 3.96 (s, 2H), 3.72 (d, *J* = 15 Hz, 2H), 1.36 (s, 6H). ^13^C {^1^H }NMR (DMSO-*d*_6_, 75 MHz): δ 170.5 (2C), 170.0 (2C), 152.2 (2C), 59.3 (2CH), 47.0 (2CH_2_), 46.3 (2C), 18.3 (2CH_3_). HMRS (ESI): *m*/*z* calcd for C_14_H_17_N_4_O_8_ [M + H]^+^, 369.1046; found, 369.1063.

#### *cis-syn*-Cyclobutane Dimer of 2-(8-Oxoguanin-9-yl)ethyl 2-(Thymin-1-yl)acetate (OG-CPD)

Compound **7** (97 mg, 0.3 mmol), EDC (102 μL, 0.6 mmol), TBTU (186 mg, 0.6 mmol), and DMAP (6.4 mg, 0.05 mmol) were dissolved in dry DMF (5 mL) and stirred at 0 °C for 30 min. Then, **4** (100 mg, 0.5 mmol) was added, and the solution was stirred for 24 h at room temperature. The unreacted **4** was filtered, and the filtrate was diluted with H_2_O (10 mL) and cooled with ice. The solid was filtered off and washed with cold water to give **OG-CPD** (50 mg, 25%) as a beige solid. ^1^H NMR (DMSO-*d*_6_, 300 MHz): δ 10.76 (s, 4H), 10.55 (s, 2H), 6.53 (s, 4H), 4.39–4.21 (m, 6H), 3.92–3.68 (m, 8H), 1.320 (s, 6H). ^13^C {^1^H }NMR (DMSO-*d*_6_, 101 MHz): δ 170.9 (2C), 169.1 (2C), 154.1 (2C), 152.9 (2C), 152.7 (2C), 151.6 (2C), 148.4 (2C), 98.8 (2C), 62.7 (2CH), 59.6 (2CH_2_), 47.4 (2CH_2_), 46.7 (2C), 38.6 (2CH_2_), 18.7 (2CH_3_). HRMS (ESI): *m*/*z* calcd for C_28_H_31_N_14_O_12_ [M + H]^+^, 755.2270; found, 755.2246.

#### *cis-syn*-Cyclobutane Dimer of 2-(Guanin-9-yl)ethyl 2-(Thymin-1-yl)acetate (G-CPD)

Compound **7** (65 mg, 0.2 mmol), EDC (34 μL, 0.4 mmol), TBTU (124 mg, 0.4 mmol), and DMAP (4.3 mg, 0.03 mmol) were dissolved in dry DMF (2 mL) and stirred at 0 °C for 30 min. Then, **2** (68 mg, 0.4 mmol) was added, and the solution was stirred for 24 h at room temperature. The reaction mixture was diluted with H_2_O (4 mL) and cooled with ice. The solid was filtered off and washed with cold water to give **G-CPD** (63 mg, 50%) as a white solid. ^1^H NMR (DMSO-*d*_6_, 300 MHz): δ 10.60 (s, 2H), 10.56 (s, 2H), 7.70 (s, 2H), 6.48 (s, 4H), 4.42–4.33 (m, 4H), 4.27–4.18 (m, 6H), 3.88–3.76 (m, 4H), 1.31 (s, 6H). ^13^C {^1^H }NMR (DMSO-*d*_6_, 75 MHz): δ 170.4 (2C), 168.5 (2C), 156.8 (2C), 153.6 (2C), 152.2 (2C), 151.2 (2C), 137.6 (2CH), 116.5 (2C), 62.9 (2CH_2_), 59.4 (2CH), 47.1 (2CH_2_), 46.2 (2C), 41.7 (2CH_2_), 18.1 (2CH_3_). HRMS (ESI): *m*/*z* calcd for C_28_H_31_N_14_O_10_ [M + H]^+^, 723.2335; found, 723.2348.

#### 2-(8-Oxoguanin-9-yl)ethyl 2-(Thymin-1-yl)acetate (**OG–T**)

Thymine-1-acetic acid (100 mg, 0.3 mmol), EDC (25.5 μL, 0.3 mmol), TBTU (93 mg, 0.3 mmol), and DMAP (4.3 mg, 0.03 mmol) were dissolved in dry DMF (2 mL) and stirred at 0 °C for 30 min. Then, **4** (51 mg, 0.3 mmol) was added, and the solution was stirred for 24 h at room temperature. The reaction mixture was diluted with H_2_O (4 mL) and cooled with ice. The solid was filtered off and washed with cold water to give **OG–T** (45 mg, 40%) as a white solid. ^1^H NMR (DMSO-*d*_6_, 300 MHz): δ 11.41 (s, 1H), 10.69 (s, 1H), 10.62 (s, 1H), 7.38 (s, 1H), 6.51 (s, 2H), 4.43 (s, 2H), 4.33 (m, 2H), 3.87 (m, 2H), 1.76 (s, 3H). ^13^C {^1^H }NMR (DMSO-*d*_6_, 75 MHz): δ 168.2 (C), 164.2 (C), 153.5 (C), 152.2 (C), 151.0 (C), 150.9 (C), 147.9 (C), 141.3 (C), 108.8 (CH), 98.3 (C), 62.4 g (CH_2_), 48.2 (CH_2_), 38.0 (CH_2_), 11.8 (CH_3_). HRMS (ESI): *m*/*z* calcd for C_14_H_16_N_7_O_6_ [M + H]^+^, 378.1162; found, 378.1166.

#### 2-(Guanin-9-yl)ethyl 2-(Thymin-1-yl)acetate (**G–T**)

Thymine acetic acid (100 mg, 0.3 mmol), EDC (25.5 μL, 0.3 mmol), TBTU (93 mg, 0.3 mmol), and DMAP (4.3 mg, 0.03 mmol) were dissolved in dry DMF (2 mL) and stirred at 0 °C for 30 min. Then, **2** (51 mg, 0.3 mmol) was added, and the solution was stirred for 24 h at room temperature. The reaction mixture was diluted with H_2_O (4 mL) and cooled with ice. The solid was filtered off and washed with cold water to give **G–T** (54 mg, 50%) as a white solid. ^1^H NMR (DMSO-*d*_6_, 300 MHz): δ 11.43 (s, 1H), 10.61 (s, 1H), 7.69 (s, 1H), 7.44 (s, 1H), 6.50 (s, 2H), 4.56–4.33 (m, 4H), 4.21 (m, 2H), 1.76 (s, 3H). ^13^C {^1^H }NMR (DMSO-*d*_6_, 75 MHz): δ 168.0 (C), 164.2 (C), 156.7 (C), 153.6 (C), 151.2 (C), 150.9 (C), 141.3 (CH), 137.5 (CH), 116.5 (C), 108.7 (C), 63.3 (CH_2_), 48.3 (CH_2_), 41.6 (CH_2_), 11.8 (CH_3_). HRMS(ESI): *m*/*z* calcd for C_14_H_17_N_7_O_5_ [M + H]^+^, 362.1099; found, 362.1100.

## Computational Details

### Quantum Chemistry Calculations

The structures of the singlet ground state of the photosensitizers, G and OG, in their neutral and cationic states, and the CPD in its neutral and anionic states were optimized using the DFT method with the B3LYP functional as implemented in the Gaussian 09 software package,^47^ with the 6-31++G** basis set and without any symmetry restriction. The standard 6-311++G(2df,p) basis set was used to calculate the energies on top of the converged geometries. Solvent effects (water) were taken into account with the polarizable continuum model approach.

### Molecular Dynamics Simulations

G-CPD and OG-CPD structures were initially generated through sketching by hand. Their geometries were optimized using the COMPASSII force field and the Materials Studio 2019 software.^48^ A water molecule was created and optimized in the same way. Subsequently, a cubic box of 20 × 20 × 20 Å was generated for each specie, creating a crystal with periodic conditions. Each box was filled with previously optimized water molecules using Monte Carlo, reaching a density of 1 g/cm^3^ (246 water molecules).

Molecular dynamic simulations were subsequently performed to investigate the most stable conformations of the two species (G-CPD and OG-CPD) in water solvent. During these simulations, the volume and temperature (298 K) were maintained constant. The total time of the simulations was 1 ns, using NVT and periodic boundary conditions with a 1 fs time step. The COMPASSII force field and the Materials Studio 2019 software were used in these computations. After the simulation, the conformations of the structures during the production dynamics were statistically analyzed.

## Data Availability

The data underlying this study are available in the published article and its Supporting Information.

## Supporting Information Available

^1^H, ^13^C, and DEPT NMR spectra for all compounds, chromatograms of steady-state photolysis of CPD and decay traces of OG-CPD at ps timescale, XYZ Cartesian coordinates for the optimized geometries of relevant compounds with the DFT method, and atom numbering for conformational analyses of the G-CPD and OG-CPD structures obtained in the molecular dynamics simulations (PDF)
