# Supplementary material for: Reductive Photocycloreversion of Cyclobutane Dimers Triggered by Guanines
Source: J Org Chem. 2023 Jul 12;88(14):10111–21. doi: 10.1021/acs.joc.3c00930 (PMC10367068; doi:10.1021/acs.joc.3c00930)
Supplement: Supplementary file 1 — jo3c00930_si_001.pdf [file jo3c00930_si_001.pdf]

Supporting Information

Reductive photocycloreversion of cyclobutane dimers triggered by guanines

Gemma M. Rodríguez-Muñiz[1], Ana B. Fraga-Timiraos[1], Miriam Navarrete-Miguel[2], Ana Borrego-Sánchez[2], Daniel Roca-Sanjuán[2], Miguel A. Miranda[1], Virginie Lhiaubet-Vallet[1,]*

[1] *Instituto Universitario Mixto de Tecnología Química (UPV-CSIC), Universitat Politècnica de València, Consejo Superior de Investigaciones Científicas, 46022 Valencia, Spain*

[2] *Instituto de Ciencia Molecular, Universitat de València, P.O.Box 22085, 46071 València, Spain*

Corresponding author's e-mail address: lvirgini@itq.upv.es

Index:

Part S1- Characterization – NMR Spectra

^{1}H, $^{13}C\{^{1}H\}$, ^{13}C DEPT-135 NMR of **1** ……………………………………**Pages S3-S4**

^{1}H, $^{13}C\{^{1}H\}$, ^{13}C DEPT-135 NMR of **2** ……………………………………**Pages S5-S6**

^{1}H, $^{13}C\{^{1}H\}$, ^{13}C DEPT-135 NMR of **3** ……………………………………**Pages S7-S8**

^{1}H, $^{13}C\{^{1}H\}$, ^{13}C DEPT-135 NMR of **4** ……………………………………**Pages S9-S10**

^{1}H, $^{13}C\{^{1}H\}$, ^{13}C DEPT-135 NMR of **5** …………………………………**Pages S11-S12**

^{1}H, $^{13}C\{^{1}H\}$, ^{13}C DEPT-135 NMR of **6** …………………………………**Pages S13-S14**

^{1}H, $^{13}C\{^{1}H\}$, ^{13}C DEPT-135 NMR of **7** …………………………………**Pages S15-S16**

^{1}H, $^{13}C\{^{1}H\}$, ^{13}C DEPT-135 NMR of **G-CPD** …………………………**Pages S17-S18**

^{1}H, $^{13}C\{^{1}H\}$, ^{13}C DEPT-135 NMR of **G-T** ……………………………**Pages S19-S20**

^{1}H, $^{13}C\{^{1}H\}$, ^{13}C DEPT-135 NMR of **OG-CPD** ………………………**Pages S21-S22**

^{1}H, $^{13}C\{^{1}H\}$, ^{13}C DEPT-135 NMR of **OG-T** ………………………… **Pages S23-S24**

Part S2- Additional Figures from experiments

Figure S1. HPLC chromatograms obtained after 0, 10, 20, 30, 40, 50, and 60 min of irradiation of **7** (0.1 mM) in PBS at pH 7.4 with 280 nm light, retention time for **7** and thymine acetic acid of 14.2 and 13.2 min, respectively………………………**Page S25**

Figure S2. Normalized fluorescence decays of **4** (black dot) and **OG-CPD** (red squares) monitored at 360 nm after excitation at 267 nm………………………………**Page S25**

Table S1. Lifetimes τ_1 and τ_2 obtained from the multiexponential fitting of the decay monitored at 360 nm of compound **4** and **OG-CPD** (see Figure S2)…………**Page S26**

Part S3- Additional Figures from computational chemistry

Table S2. XYZ Cartesian coordinates (in A) for the geometries of relevant compounds optimized with the DFT method, the B3LYP functional and the 6-31++G** basis set…………………………………………………………………**Pages S27-S31**

Figure S3. Atom numbering related to the G-CPD structures displayed in Figure 5 ……………………………………………………………………………**Page S32**

Figure S4. Atom numbering related to the OG-CPD structures displayed in Figure 6 ……………………………………………………………………………… **Page S32**

Part S1

^{1}H (300 MHz), ^{13}C{^{1}H }(75 MHz), ^{13}C DEPT-135 NMR of **1** in DMSO-d$_6$

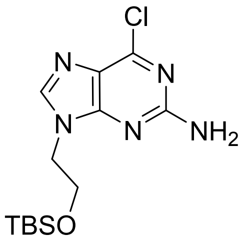

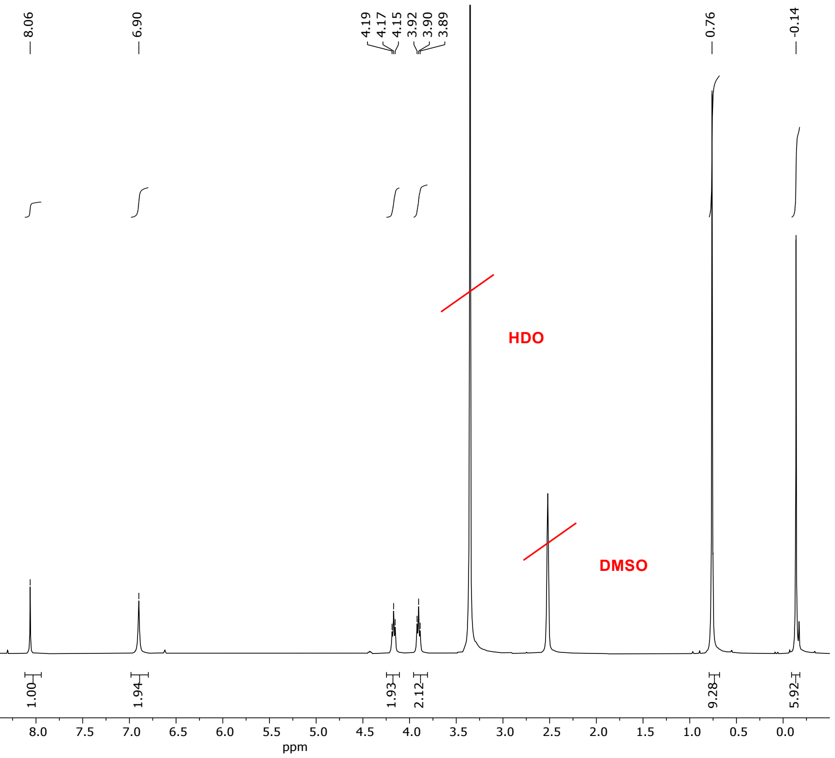

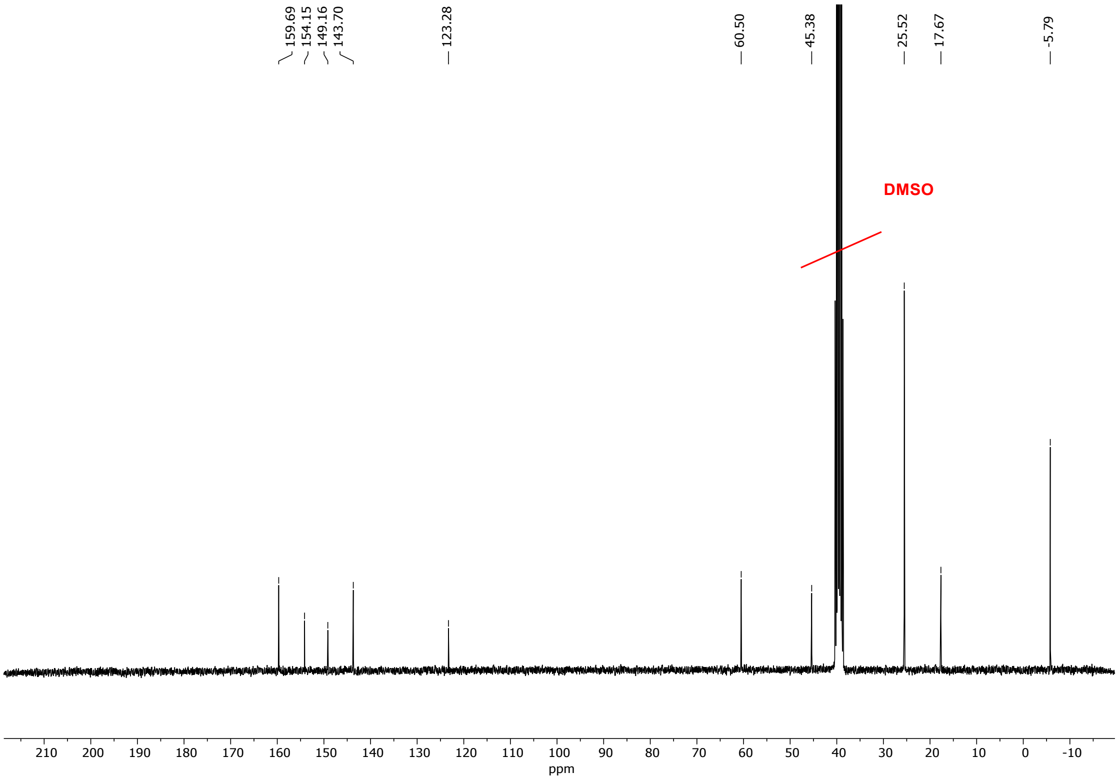

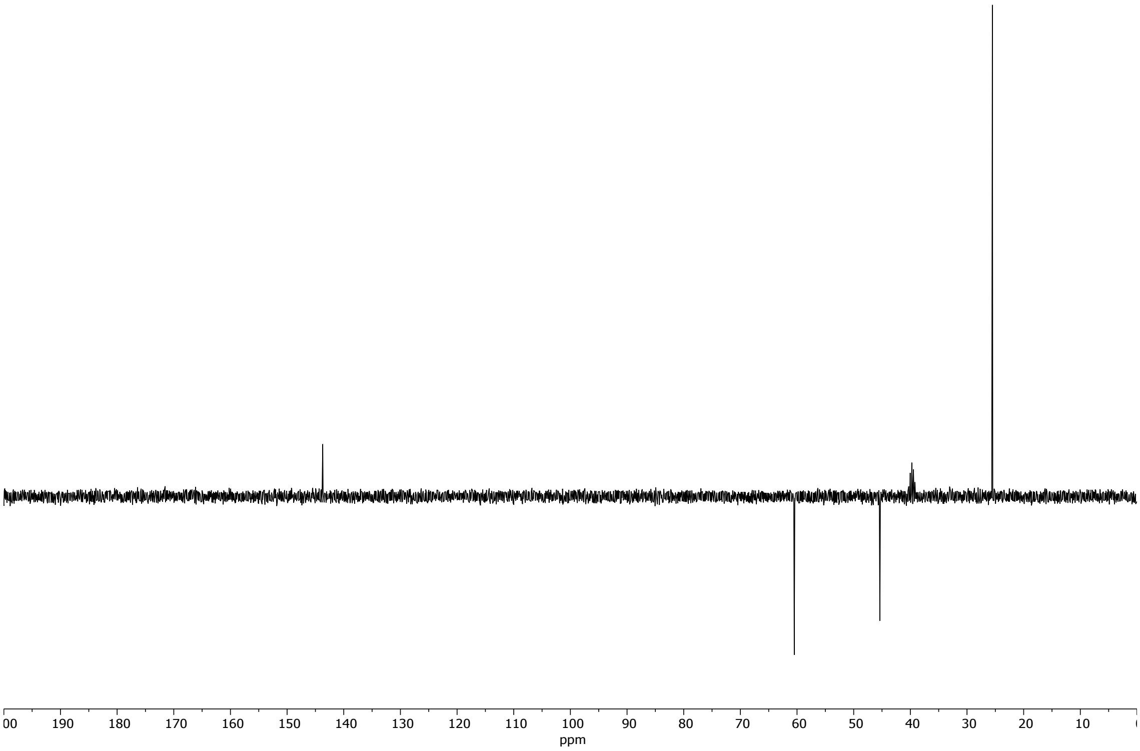
00 190 180 170 160 150 140 130 120 110 100 90 80 70 60 50 40 30 20 10
ppm

^{1}H (300 MHz), ^{13}C {^{1}H }(75 MHz), ^{13}C DEPT-135 NMR of **2** in DMSO-d_6

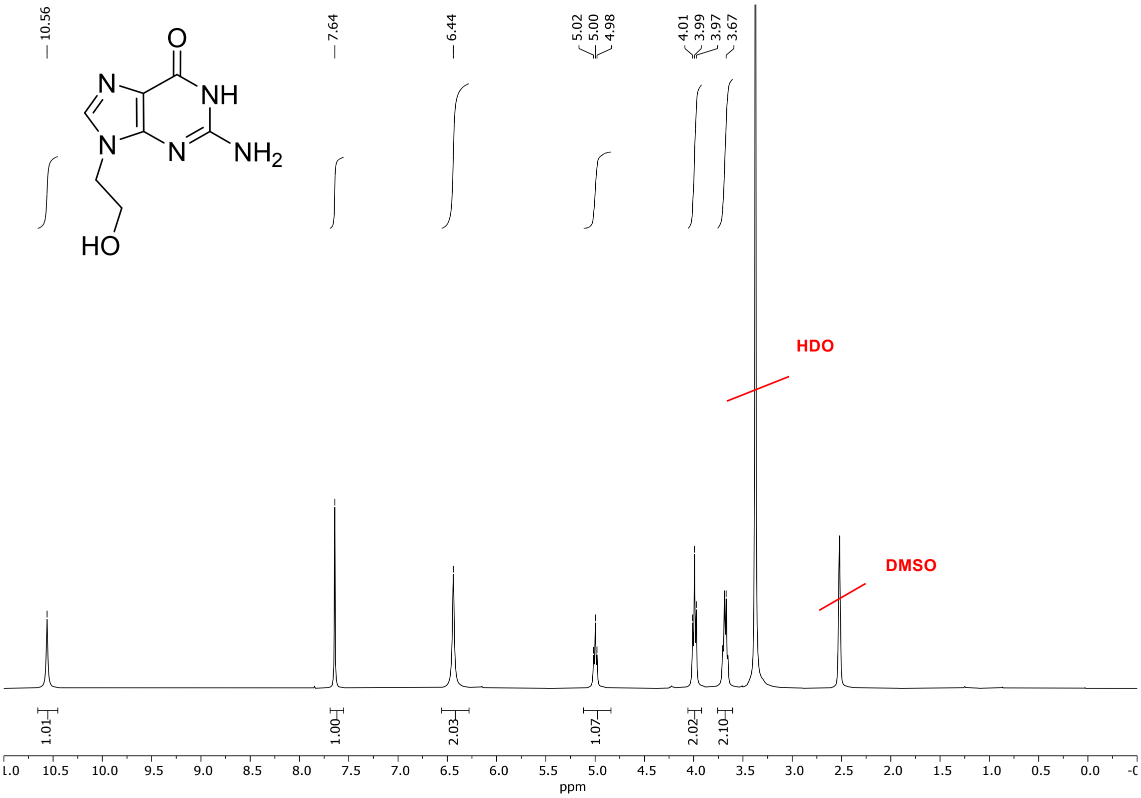

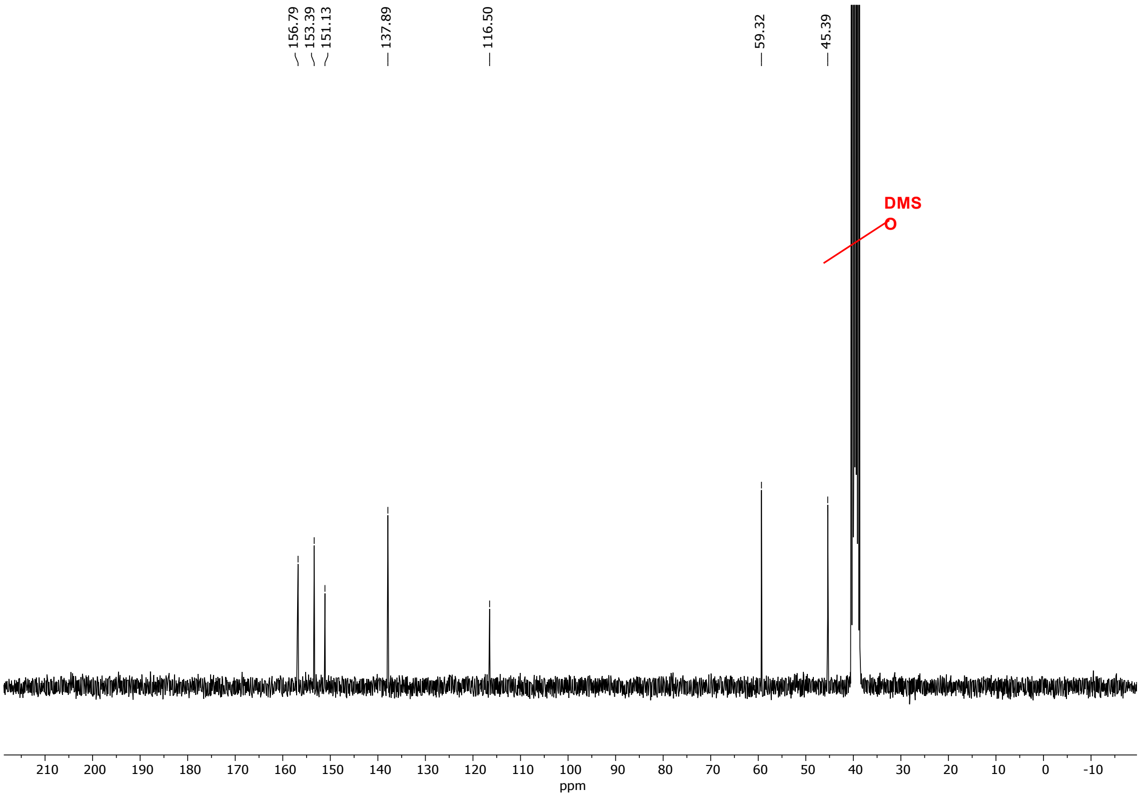

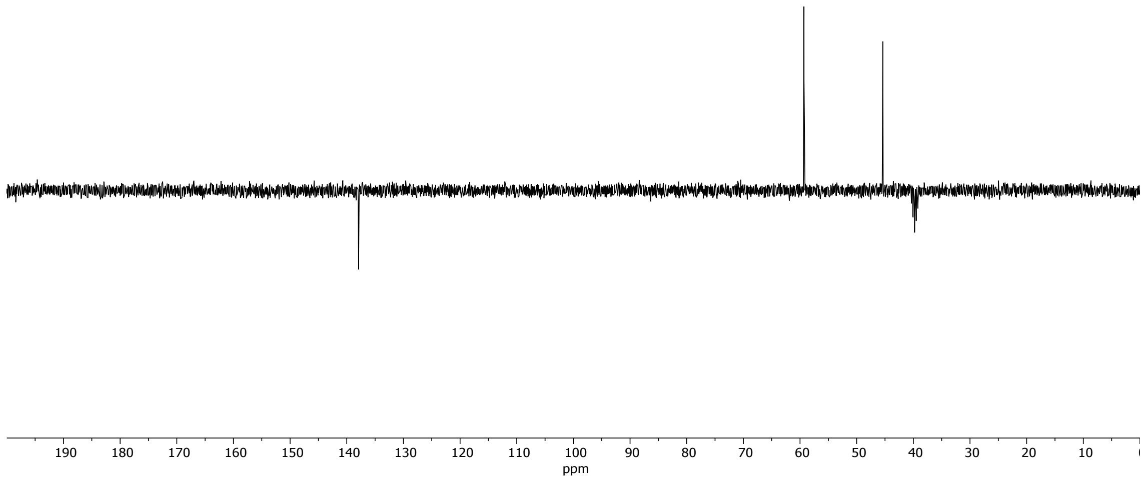
190 180 170 160 150 140 130 120 110 100 90 80 70 60 50 40 30 20 10
ppm

^{1}H (300 MHz), ^{13}C {^{1}H }(75 MHz), ^{13}C DEPT-135 NMR of **3** in DMSO-d_6

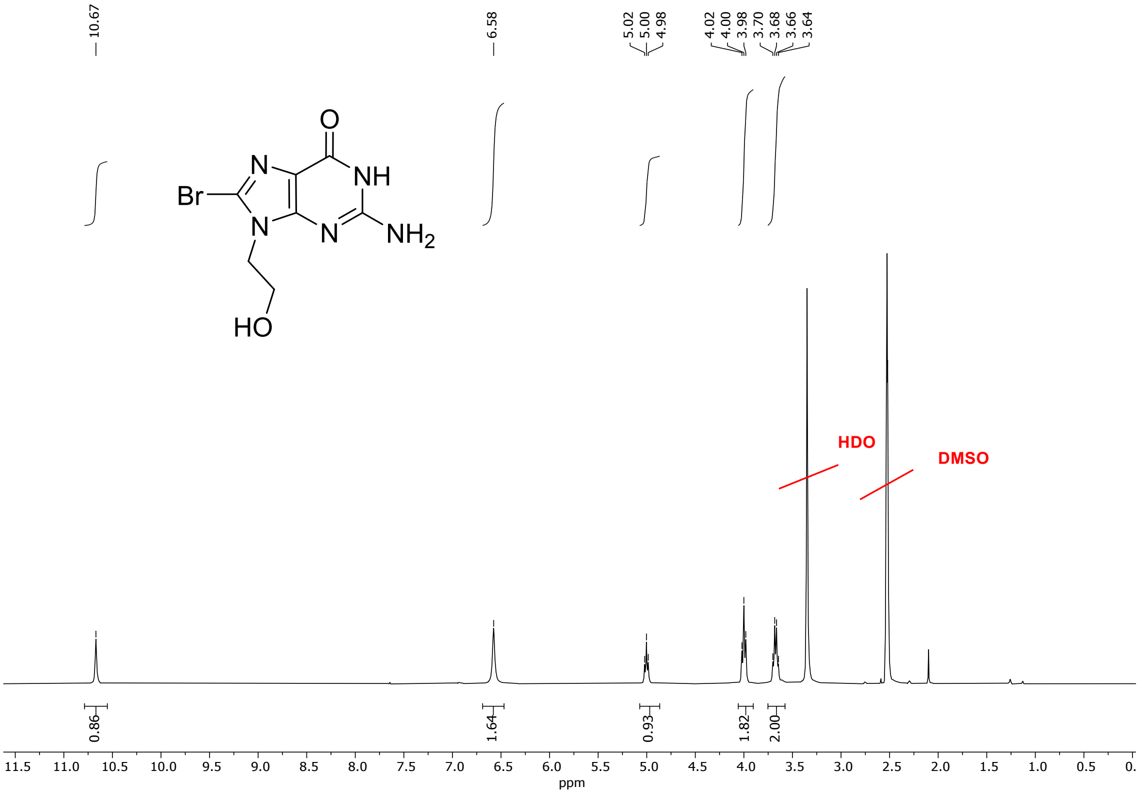

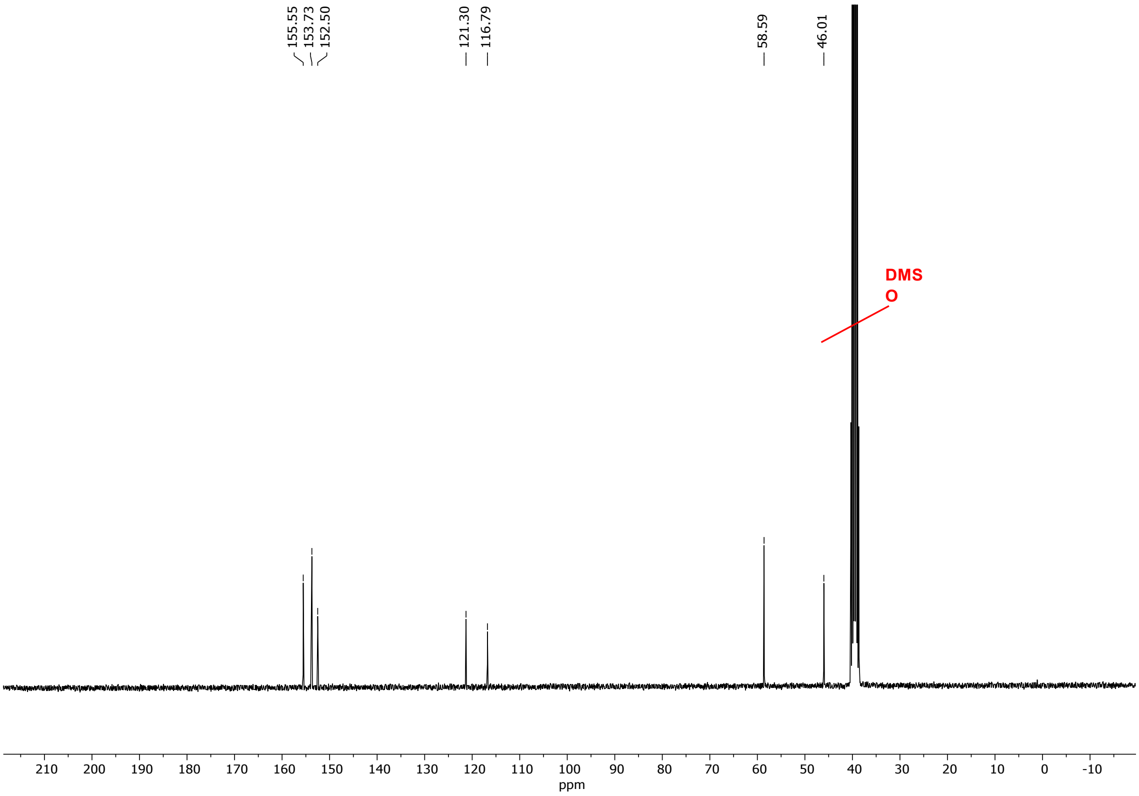

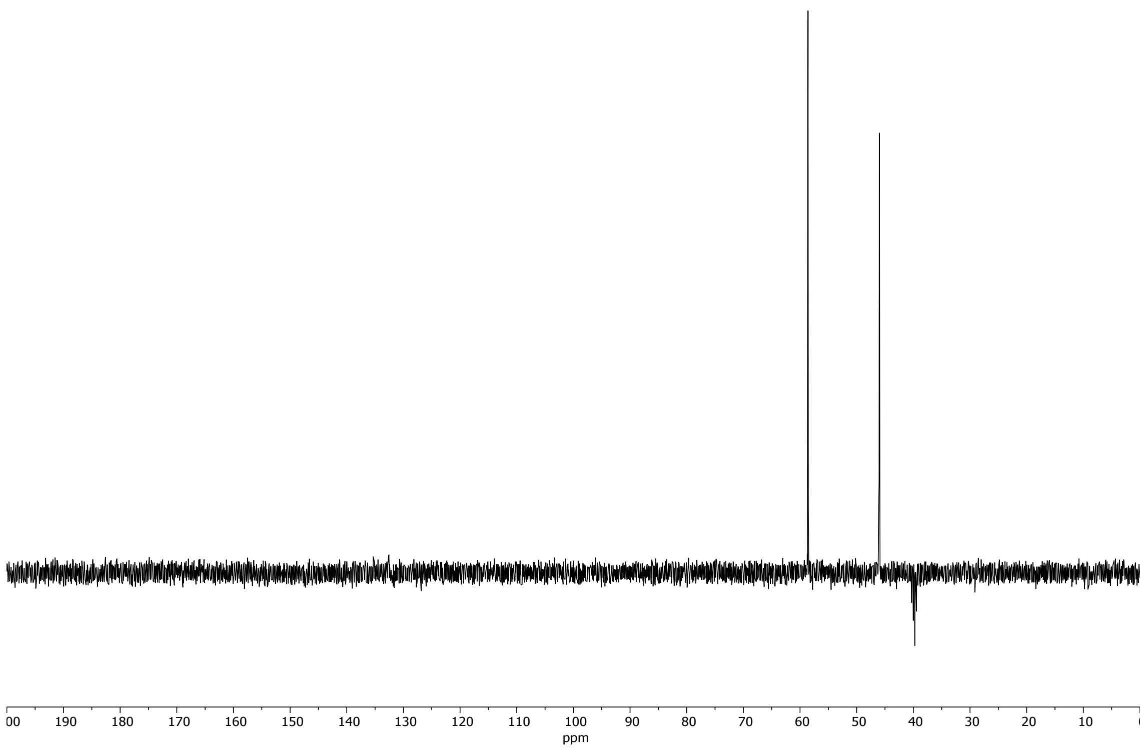
00
190
180
170
160
150
140
130
120
110
100
90
80
70
60
50
40
30
20
10
ppm

^{1}H (300 MHz), ^{13}C {^{1}H }(75 MHz), ^{13}C DEPT-135 NMR of **4** in DMSO-d$_6$

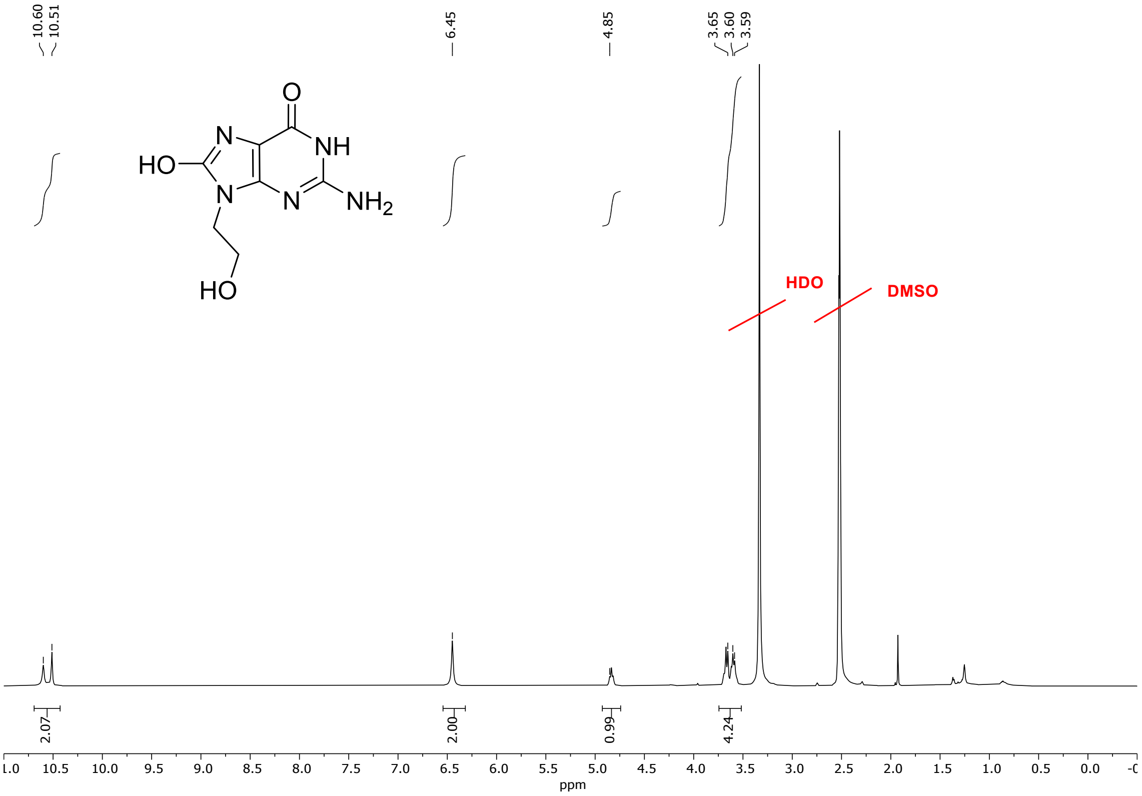

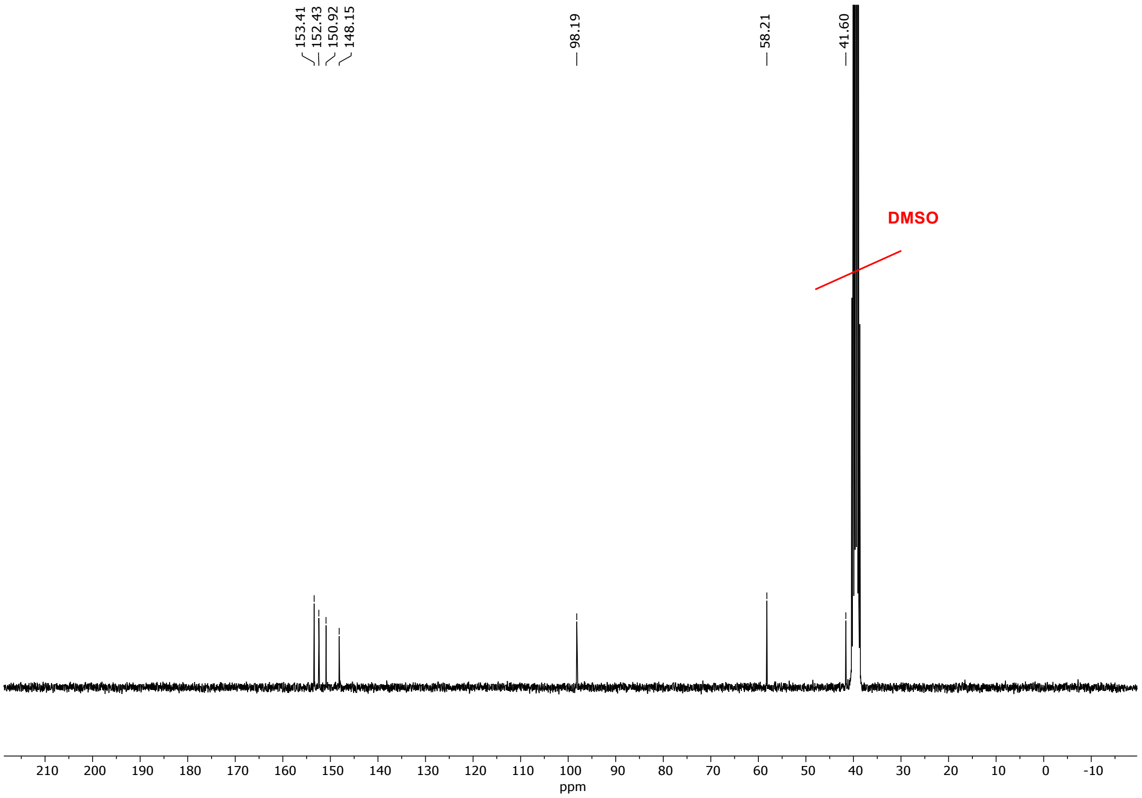

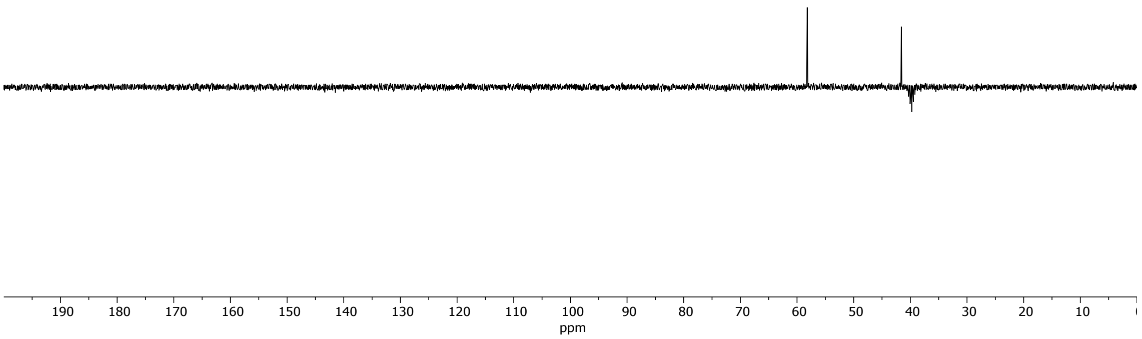
190 180 170 160 150 140 130 120 110 100 90 80 70 60 50 40 30 20 10
ppm

^{1}H (300 MHz), ^{13}C {^{1}H }(75 MHz), ^{13}C DEPT-135 NMR of **5** in DMSO-d_6

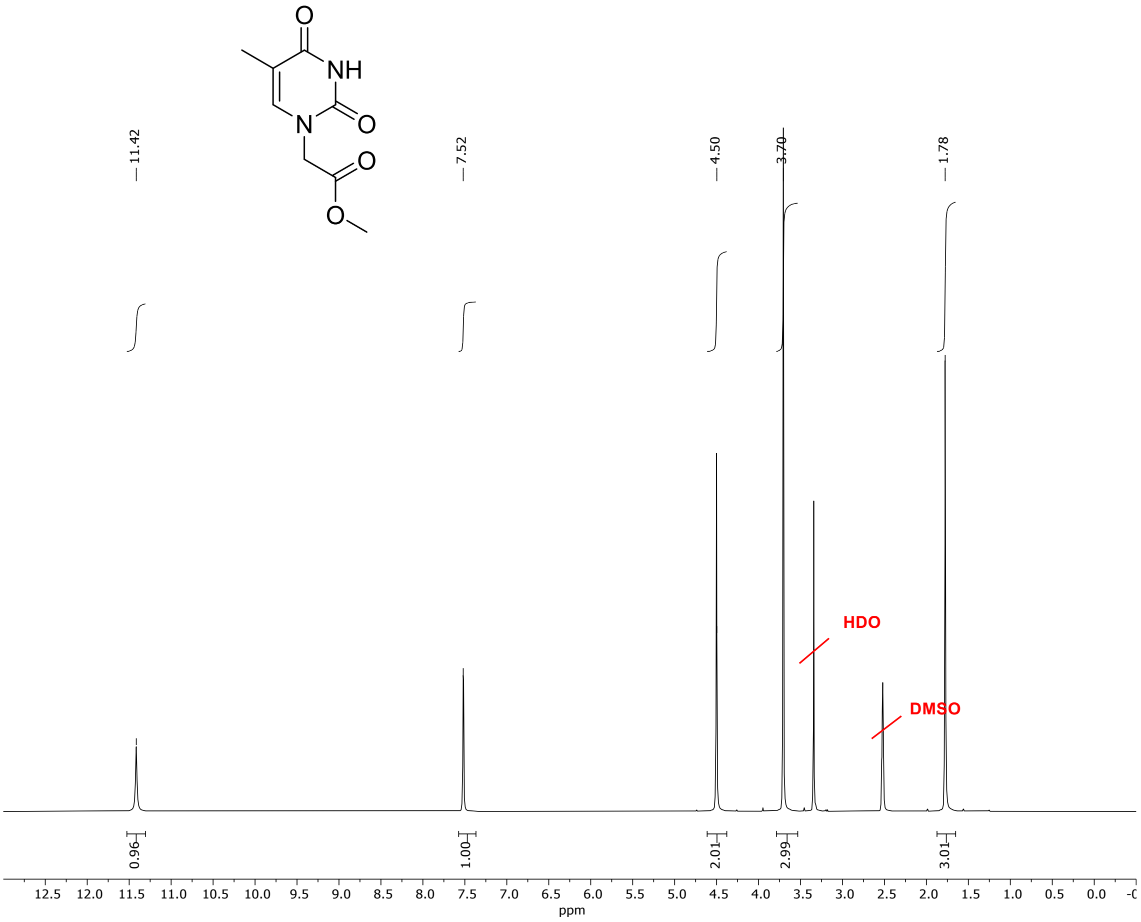

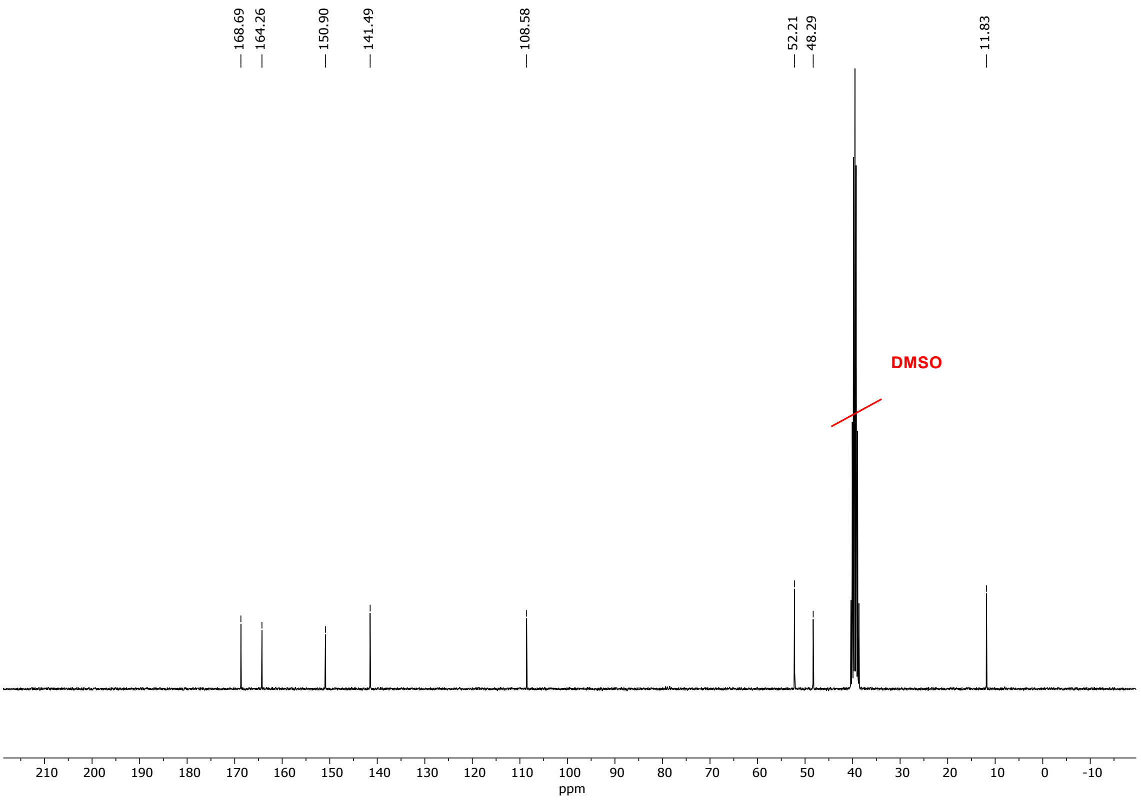

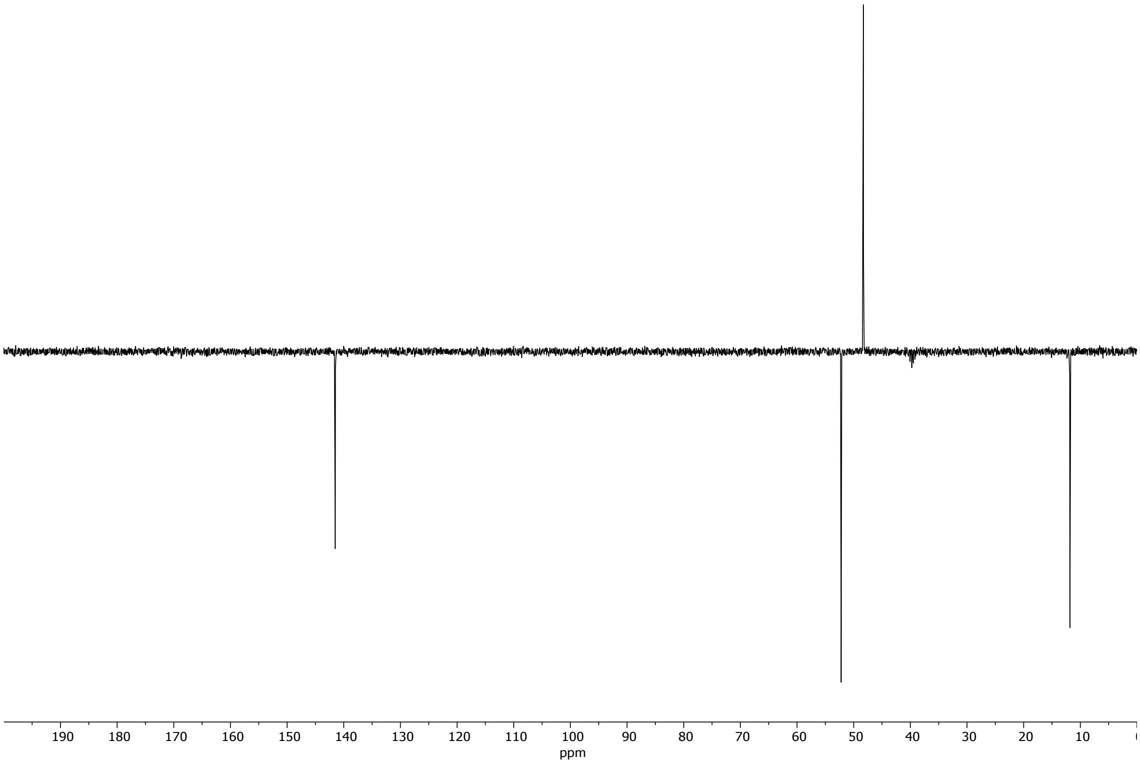
190
180
170
160
150
140
130
120
110
100
90
80
70
60
50
40
30
20
10
ppm

^{1}H (300 MHz), ^{13}C {^{1}H }(75 MHz), ^{13}C DEPT-135 NMR of **6** in DMSO-d_6

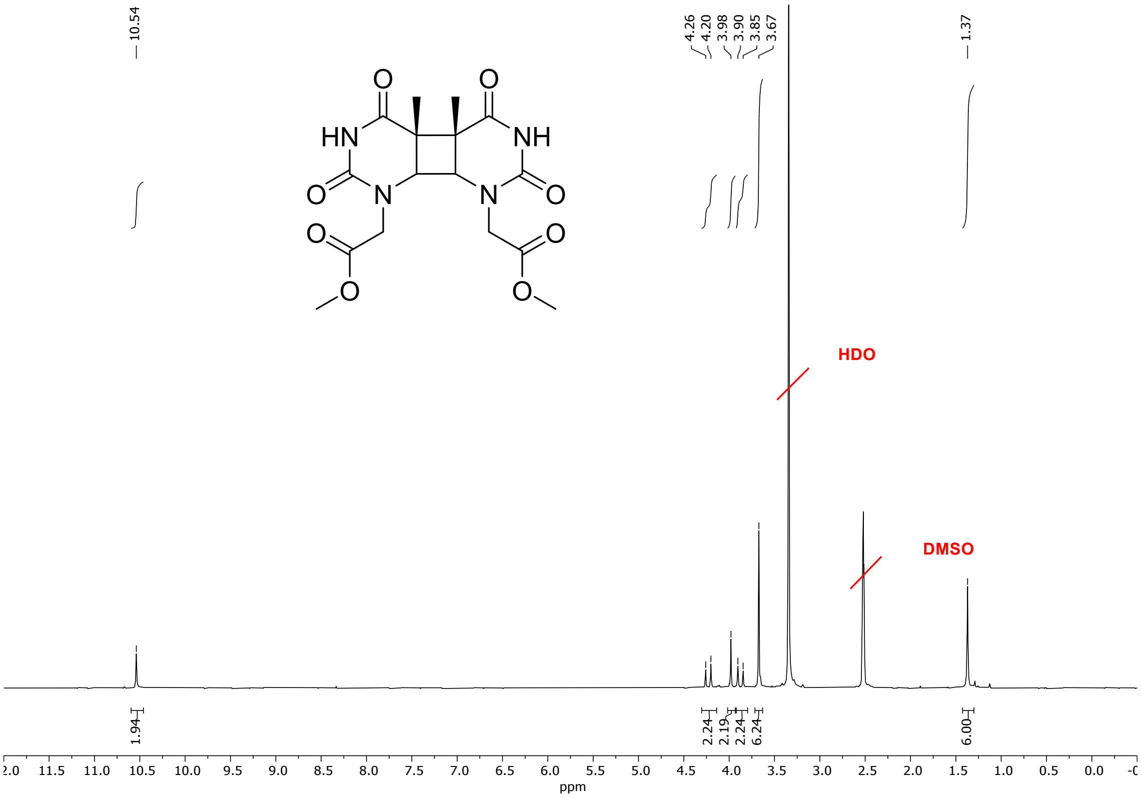

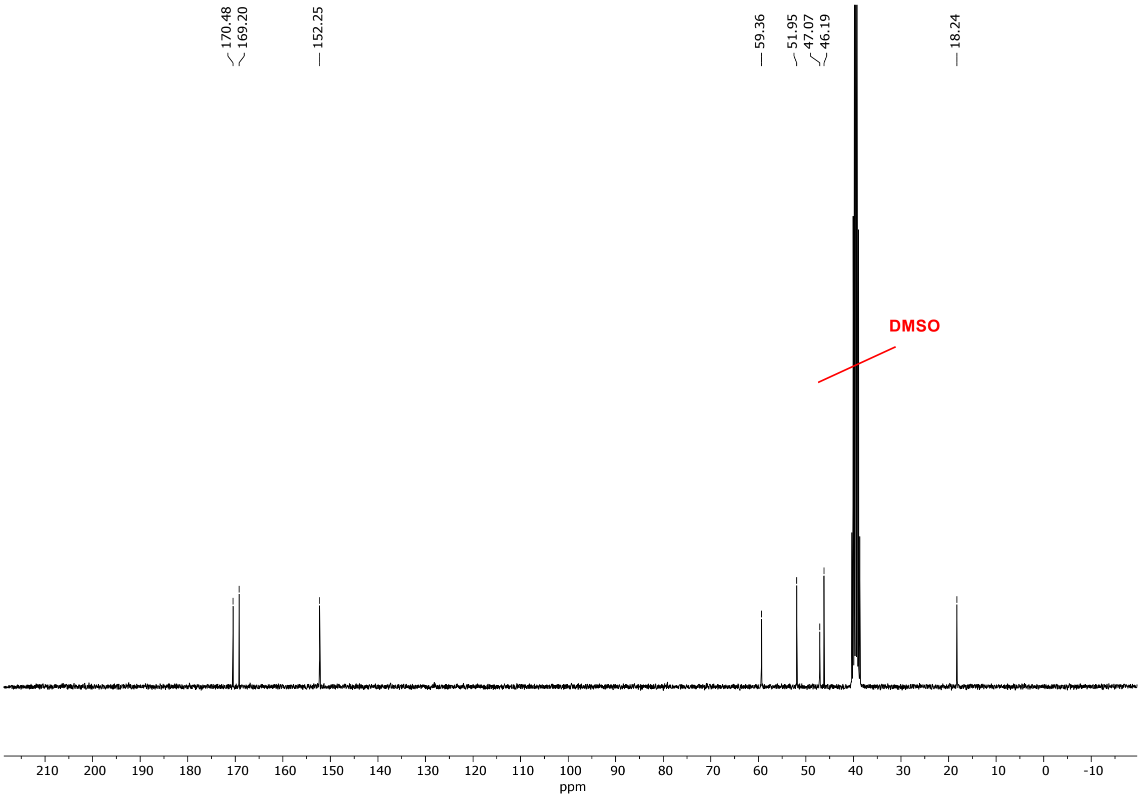

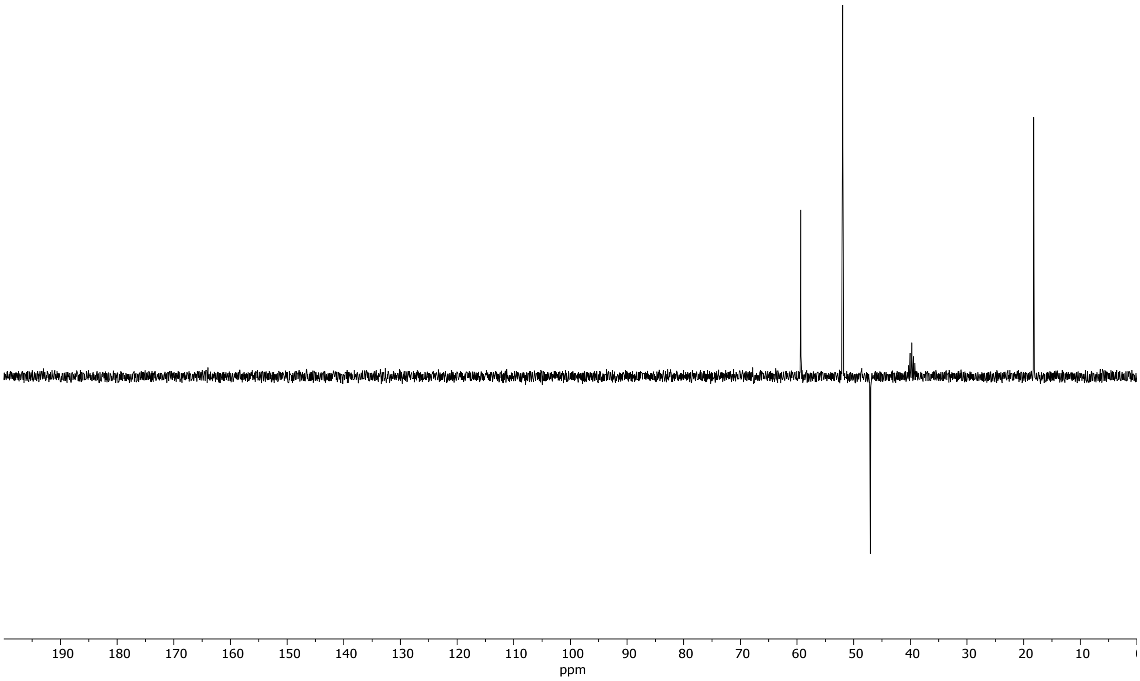
190 180 170 160 150 140 130 120 110 100 90 80 70 60 50 40 30 20 10
ppm

^{1}H (300 MHz), ^{13}C {^{1}H }(75 MHz), ^{13}C DEPT-135 NMR of **7** in DMSO-d_6

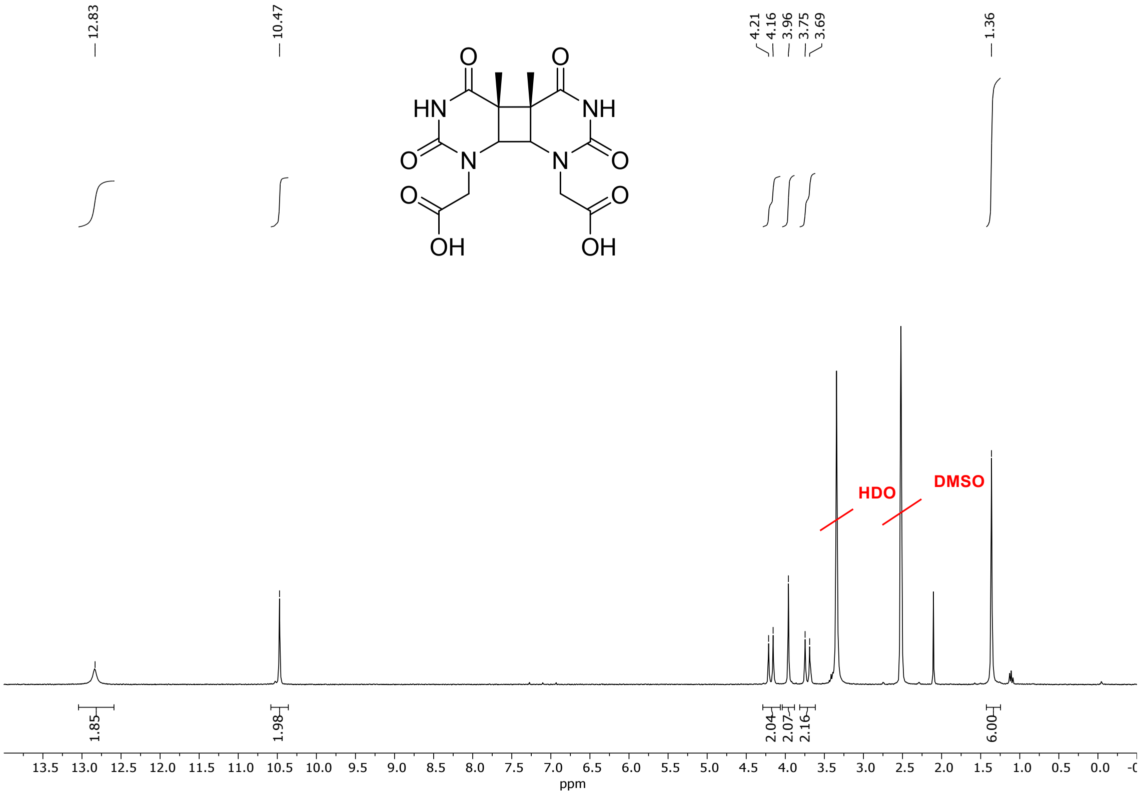

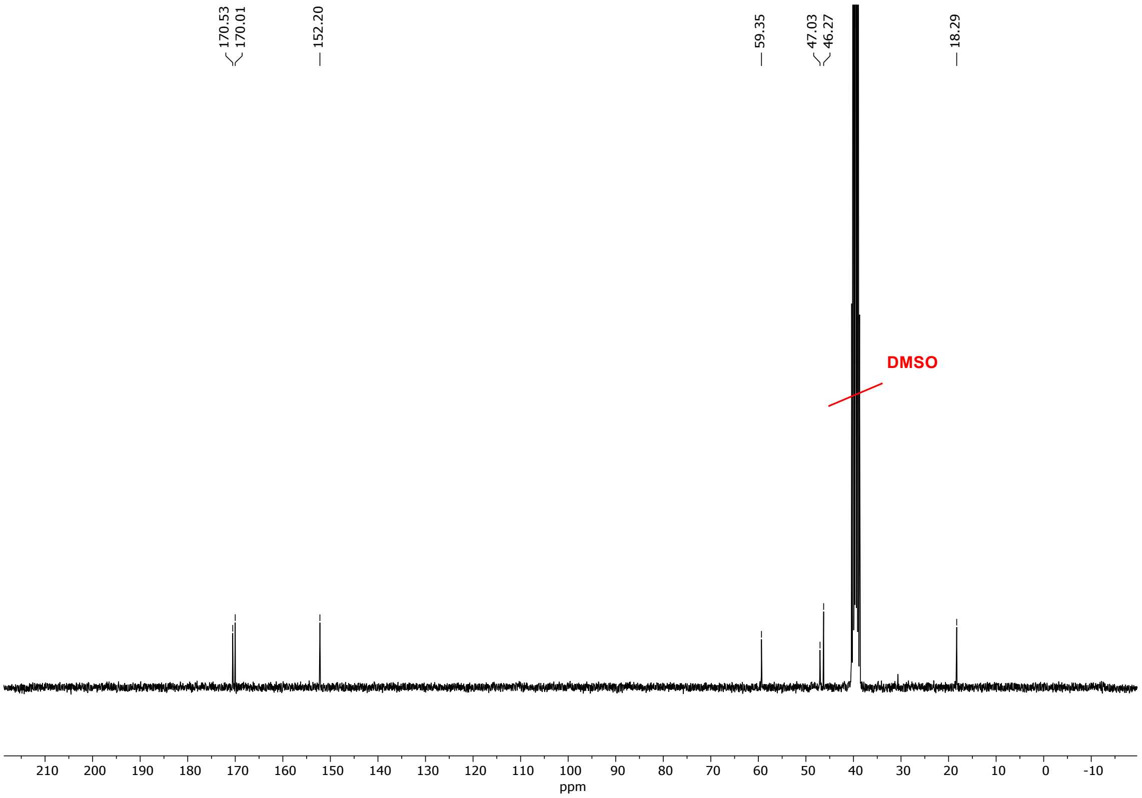

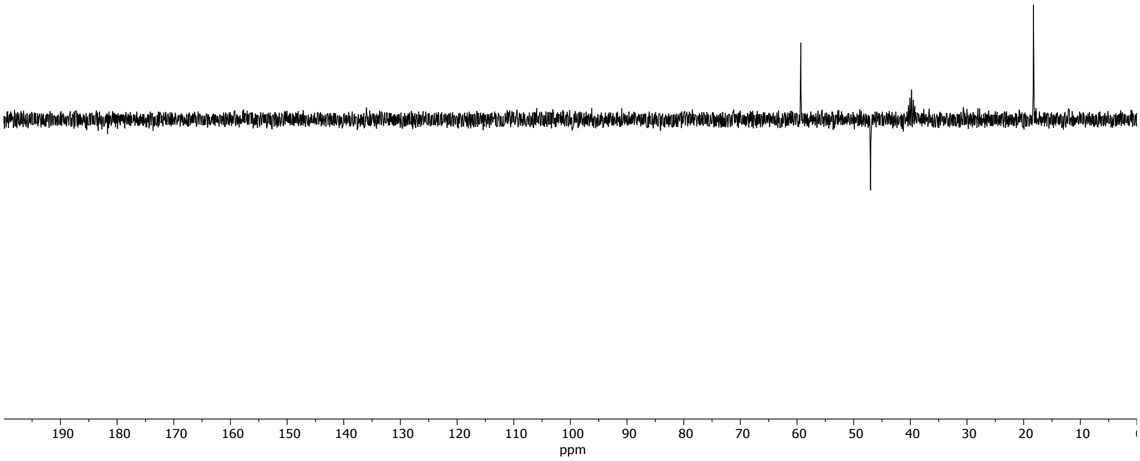
190 180 170 160 150 140 130 120 110 100 90 80 70 60 50 40 30 20 10
ppm

^{1}H (300 MHz), ^{13}C {^{1}H }(75 MHz), ^{13}C DEPT-135 NMR of **G-CPD** in DMSO-d_6

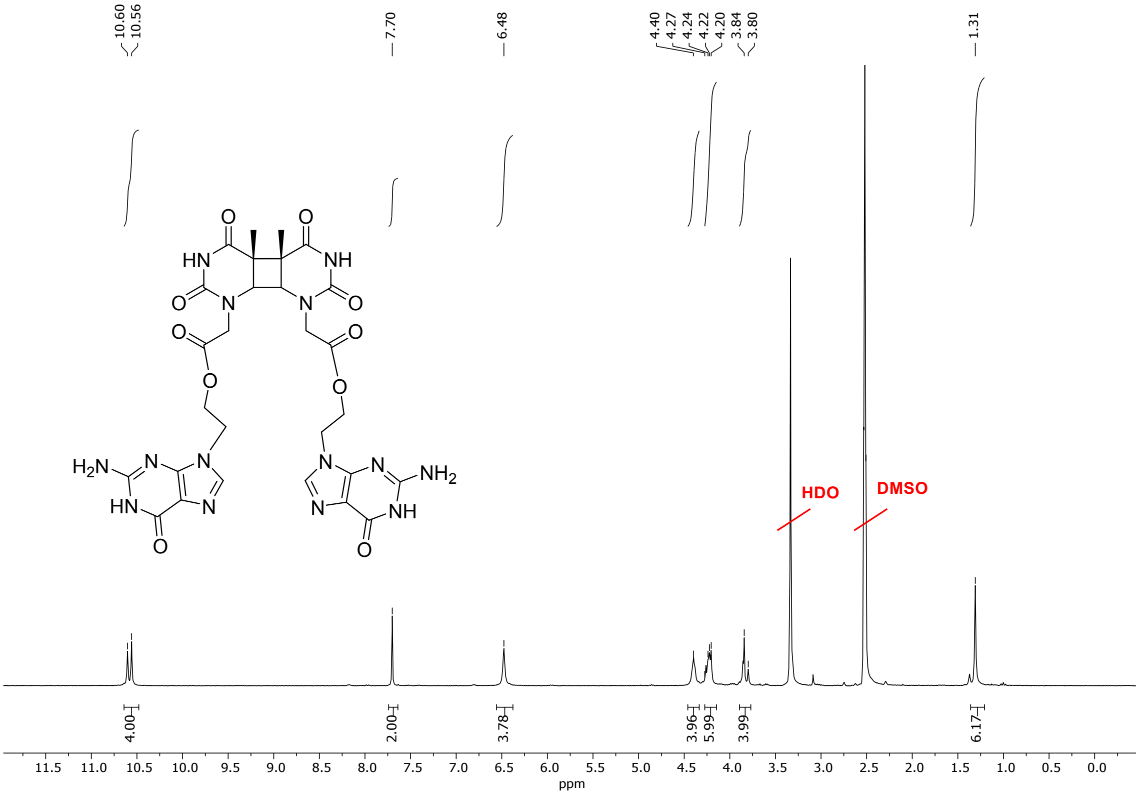

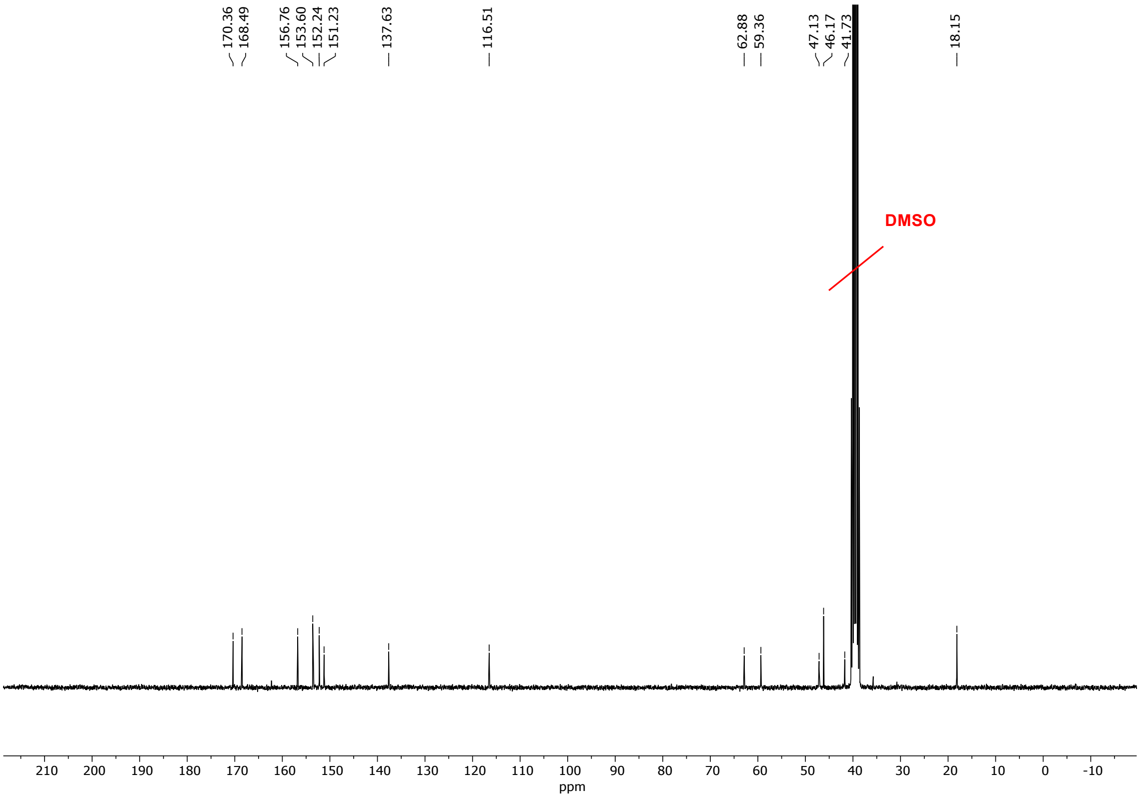

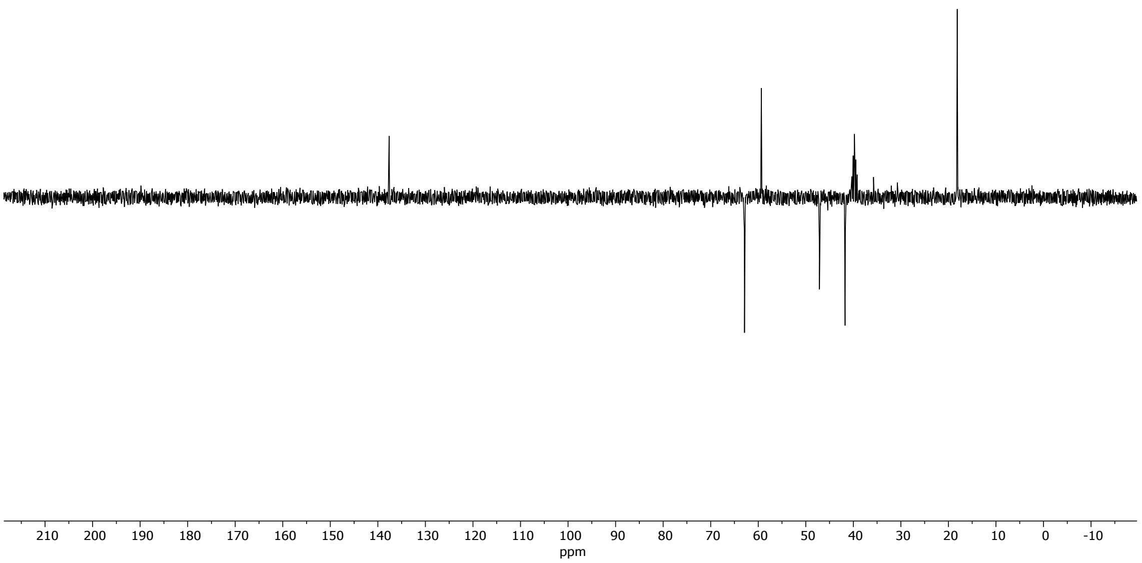
210 200 190 180 170 160 150 140 130 120 110 100 90 80 70 60 50 40 30 20 10 0 -10
ppm

^{1}H (300 MHz), ^{13}C {^{1}H }(75 MHz), ^{13}C DEPT-135 NMR of **G-T** in DMSO-d_6

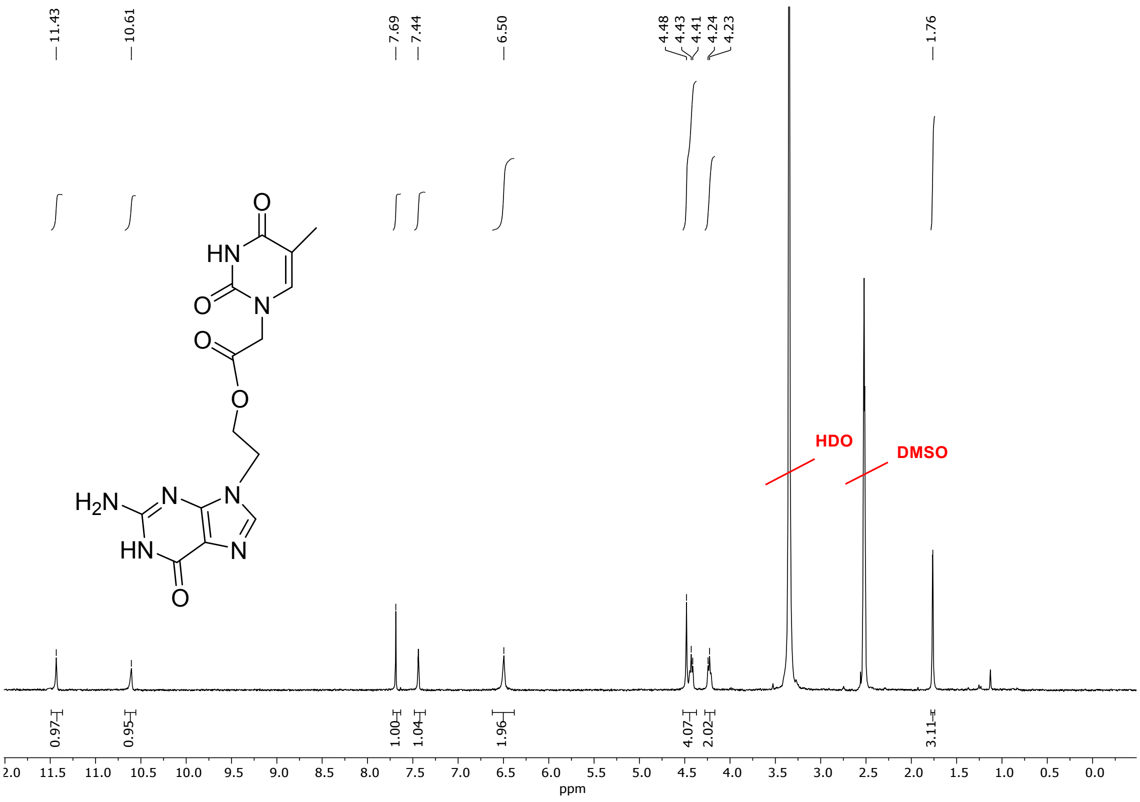

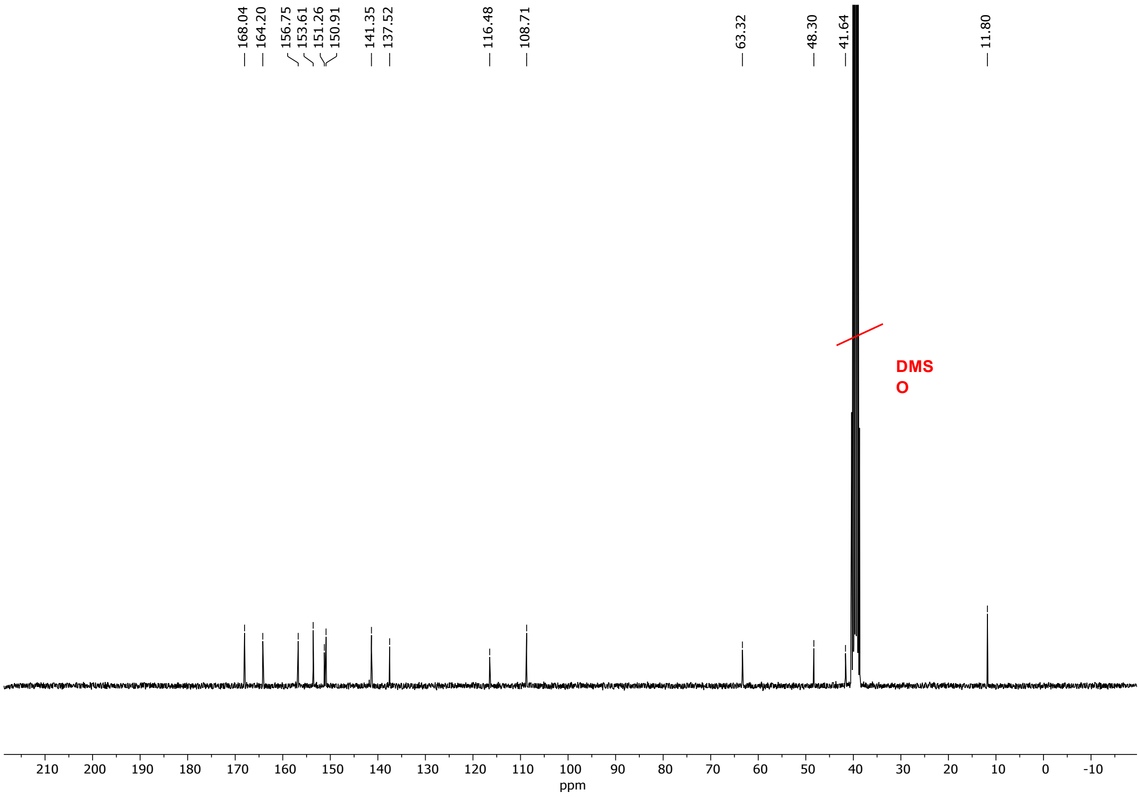

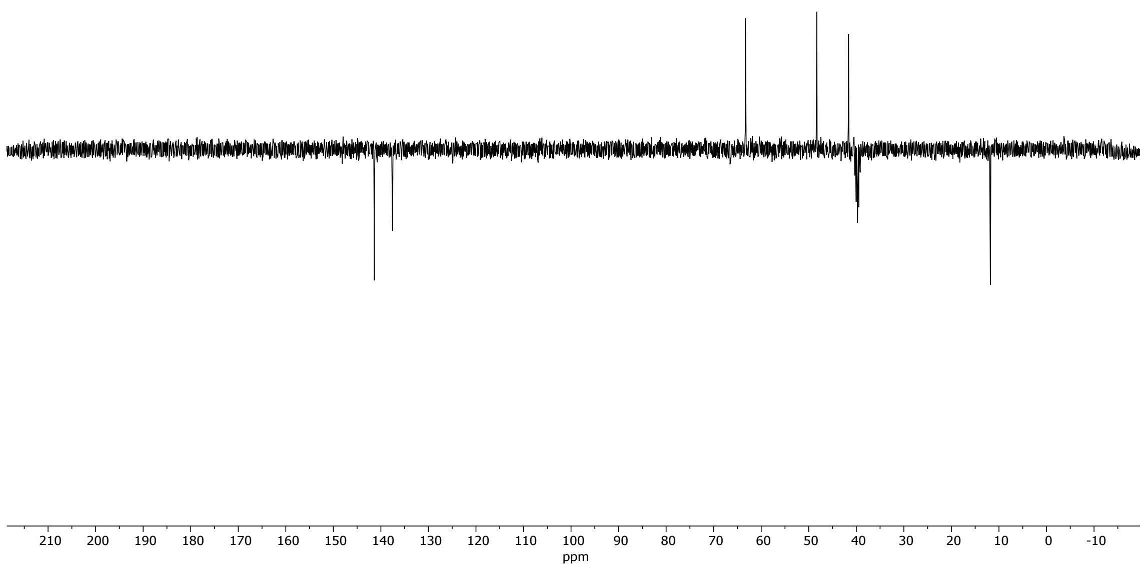
210 200 190 180 170 160 150 140 130 120 110 100 90 80 70 60 50 40 30 20 10 0 -10
ppm

^{1}H (300 MHz), ^{13}C {^{1}H }(101 MHz), ^{13}C DEPT-135 NMR of **OG-CPD** in DMSO-d_6

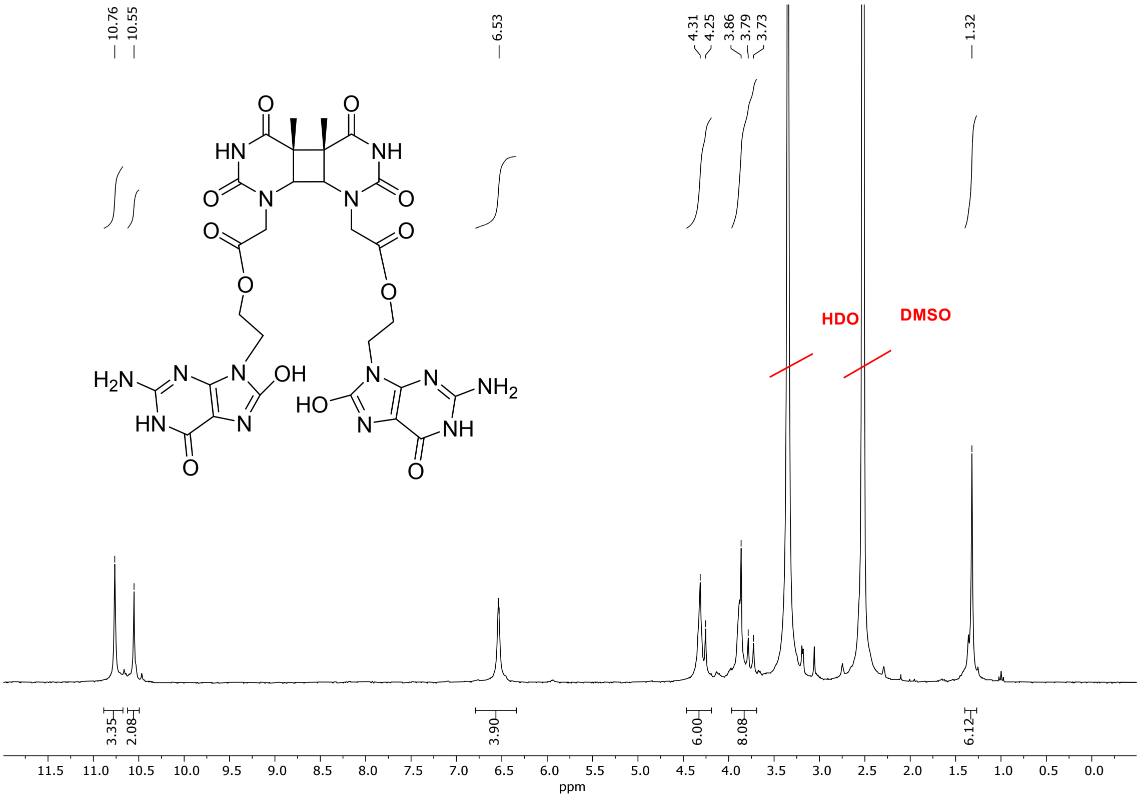

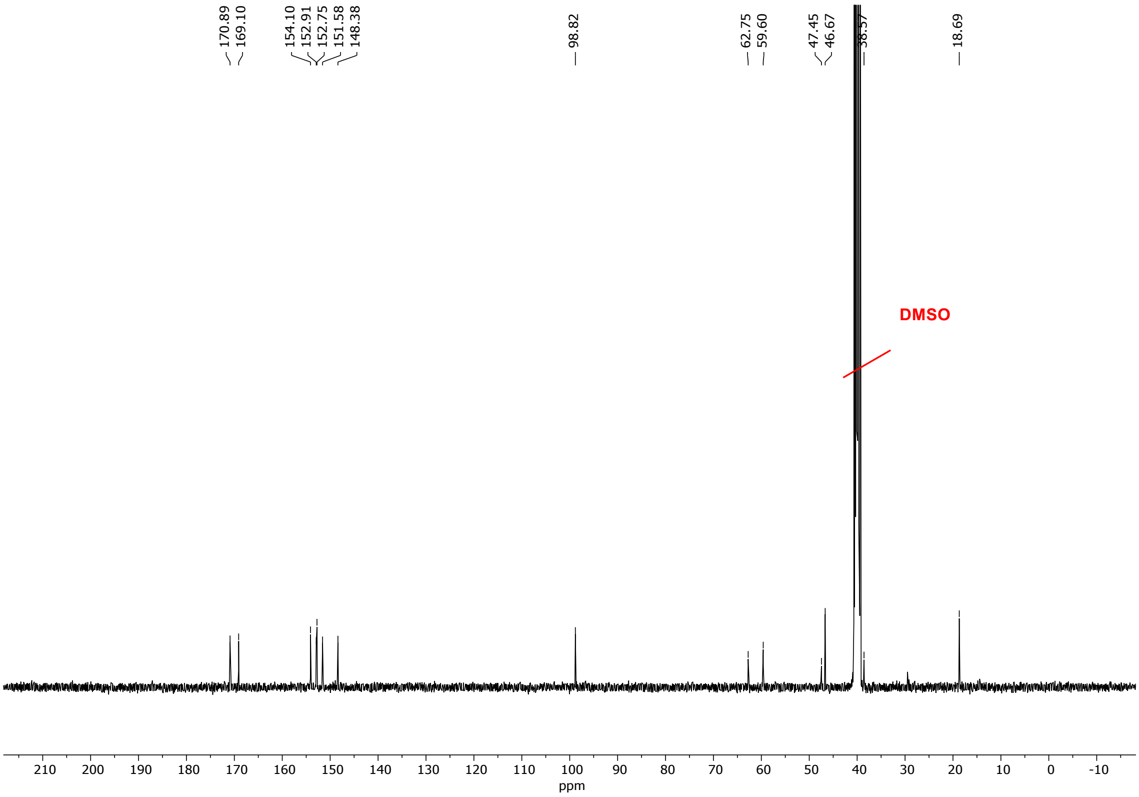

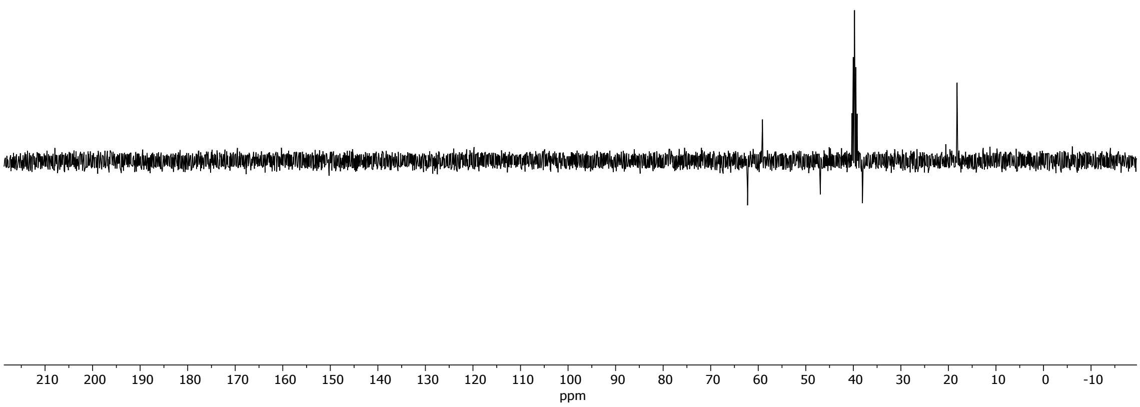

210 200 190 180 170 160 150 140 130 120 110 100 90 80 70 60 50 40 30 20 10 0 -10
ppm

^{1}H (300 MHz), ^{13}C {^{1}H }(75 MHz), ^{13}C DEPT-135 NMR of **OG-T** in DMSO-d_6

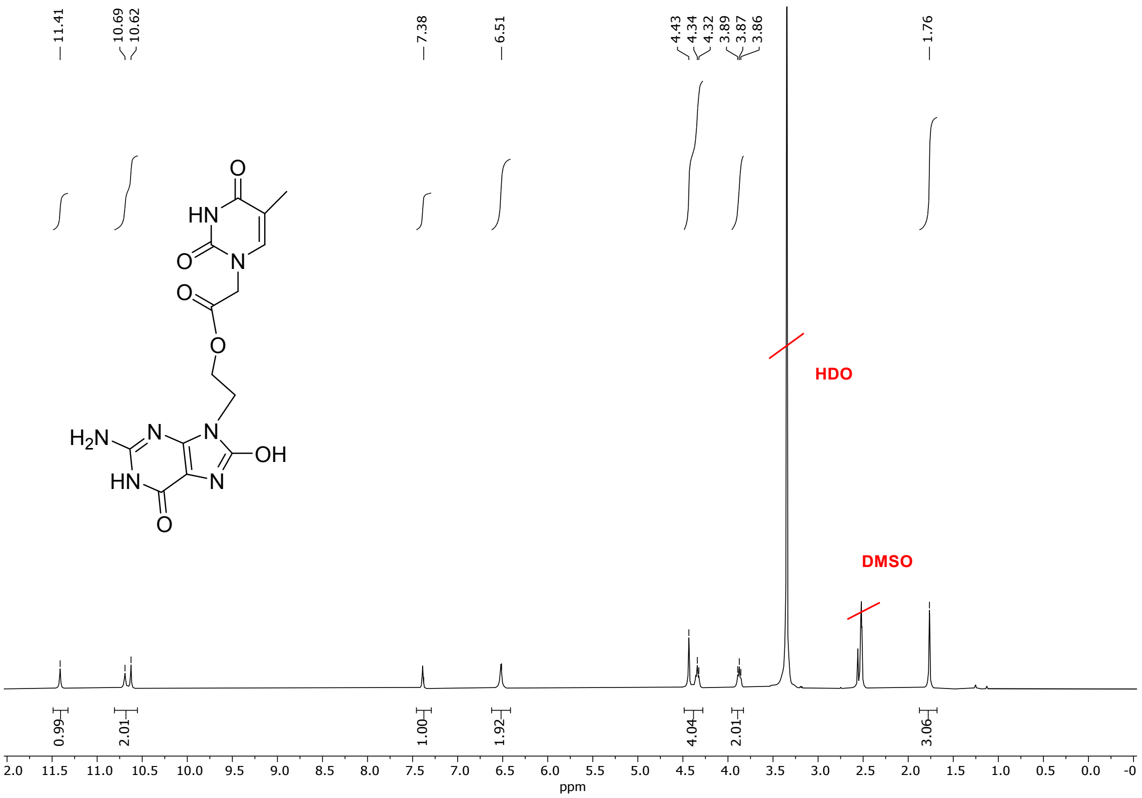

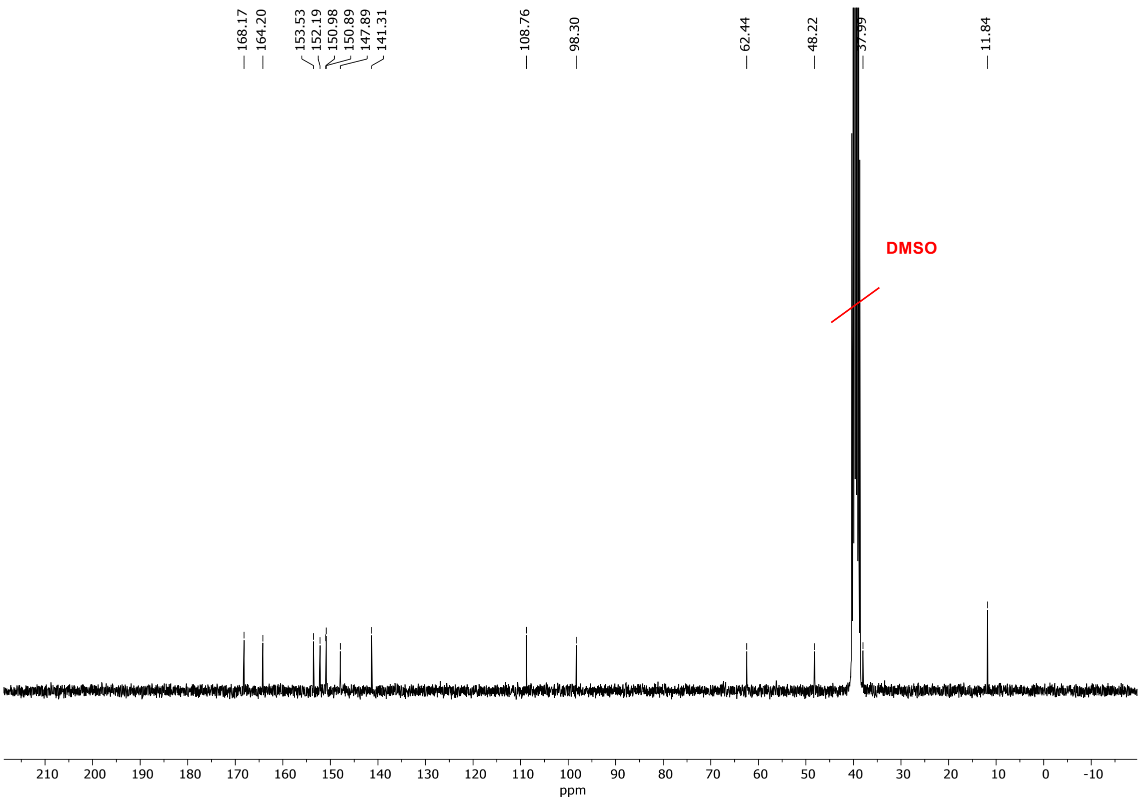

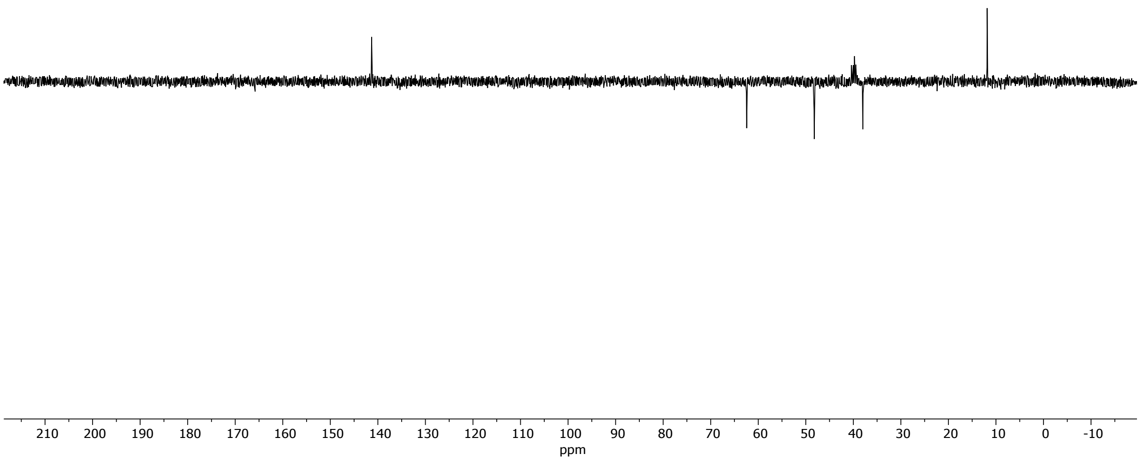
210 200 190 180 170 160 150 140 130 120 110 100 90 80 70 60 50 40 30 20 10 0 -10
ppm

Part S2

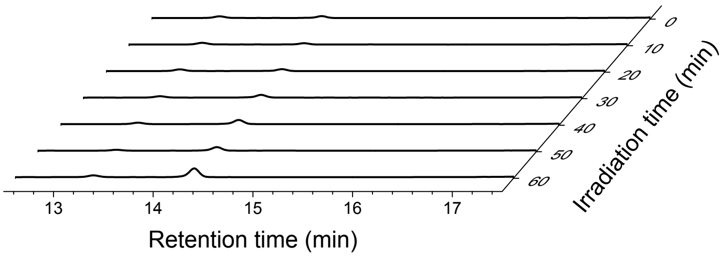

Figure S1. HPLC chromatograms obtained after 0, 10, 20, 30, 40, 50, and 60 min of irradiation of **7** (0.1 mM) in PBS at pH 7.4 with 280 nm light, retention time for **7** and thymine acetic acid of 14.2 and 13.2 min, respectively.

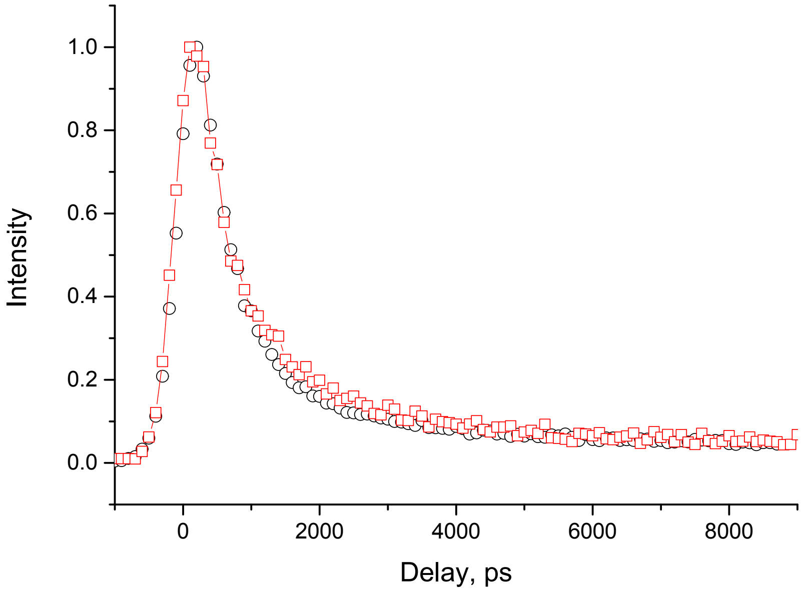

Figure S2. Normalized fluorescence decays of **4** (black dot) and **OG-CPD** (red squares) monitored at 360 nm after excitation at 267 nm

Table S1. Lifetimes τ_1 and τ_2 obtained from the multiexponential fitting of the decay monitored at 360 nm of compound **4** and **OG-CPD** (see Figure S2)

	τ_1 (ps) (a_1)	τ_2 (ps) (a_2)	$<\tau>^a$ (ps)
Compound **4**	0.26 (0.61)	0.87 (0.34)	0.45
**OG-CPD**	0.16 (0.64)	1.0 (0.32)	0.42

$^a <\tau> = a_1\tau_1 + a_2\tau_2$,

Part S3

Table S2. XYZ Cartesian coordinates (in A) for the geometries of relevant compounds optimized with the DFT method, the B3LYP functional and the 6-31+G* basis set.

17
Min S_0 OG Neutral H_2O, E=-618.051103070 a.u., 0 imaginary frequencies

C	-0.395071	0.607557	0.000005
C	0.743215	1.446829	0.000008
C	-0.245692	-0.763123	0.000002
N	-1.763537	0.878266	0.000000
N	-1.508651	-1.301099	0.000000
N	0.904321	-1.461068	0.000000
N	1.923072	0.672182	-0.000001
C	-2.473696	-0.293856	0.000005
H	-1.737768	-2.285512	-0.000003
H	-2.198120	1.789175	-0.000004
C	1.981306	-0.695481	-0.000002
H	2.783530	1.209908	-0.000010
N	3.198736	-1.267523	-0.000014
H	4.052568	-0.733331	0.000032
H	3.253508	-2.274094	0.000016
O	-3.690380	-0.447162	-0.000001
O	0.803920	2.676787	-0.000003

17
Min S_0 OG Cation H_2O, E=-617.849706721 a.u., 0 imaginary frequencies

C	0.000000	0.734337	0.000000
C	-1.444030	0.798662	0.000000
C	0.660449	-0.536231	0.000000
N	0.945071	1.690865	0.000000
N	1.996390	-0.274636	0.000000
N	0.069342	-1.707689	0.000000
N	-2.000411	-0.494477	0.000000
C	2.233671	1.104728	0.000000
H	2.740831	-0.964429	0.000000
H	0.796594	2.696054	0.000000
C	-1.284566	-1.663544	0.000000
H	-3.017992	-0.525261	0.000000
N	-1.933640	-2.818023	0.000000
H	-2.944168	-2.887700	0.000000
H	-1.396358	-3.677849	0.000000
O	3.294578	1.686633	0.000000
O	-2.133242	1.808266	0.000000

16
Min S_0 G Neutral H_2O, E=-542.769807426 a.u., 0 imaginary frequencies

C	0.846024	0.502068	0.000002
C	-0.222983	1.450979	0.000006
C	0.530524	-0.860281	0.000001
N	2.219984	0.689989	-0.000001
N	1.741298	-1.500785	0.000002
N	-0.689816	-1.443274	0.000000
N	-1.478625	0.795011	0.000000
C	2.717342	-0.525250	-0.000001
C	-1.682228	-0.568379	-0.000002
H	-2.279816	1.418753	-0.000004
N	-2.961827	-1.005418	-0.000011
H	-3.757165	-0.383037	0.000021
H	-3.133123	-2.001054	0.000012
O	-0.179815	2.687833	0.000000
H	1.890686	-2.502603	0.000003
H	3.768760	-0.778210	-0.000002

16

Min S_0 G Cation H_2O, E=-542.557498673 a.u., 0 imaginary frequencies

C	-0.846780	0.518175	0.000000
C	0.257751	1.469039	0.000000
C	-0.533893	-0.884589	0.000000
N	-2.170778	0.722038	-0.000001
N	-1.762217	-1.498685	0.000000
N	0.652373	-1.443840	0.000001
N	1.503873	0.802068	0.000001
C	-2.698854	-0.514238	0.000000
C	1.683889	-0.552941	0.000001
H	2.314190	1.417878	0.000000
N	2.906643	-1.057624	0.000000
H	3.748404	-0.491557	0.000000
H	3.012480	-2.066754	0.000000
O	0.192590	2.686090	-0.000001
H	-1.939173	-2.498703	0.000000
H	-3.758547	-0.729958	-0.000001

30

Min S_0 CPD Neutral Gas Phase, E=-908.564188905 a.u., 0 imaginary frequencies

N	2.210863	-0.269597	-1.100059
C	2.732046	0.238002	0.060397
N	1.906517	0.105860	1.187550
C	0.653217	-0.476260	1.283562
C	0.182782	-1.275763	0.063181
C	0.271447	-2.758728	0.442459
C	0.883614	-0.810278	-1.241182
C	-1.984021	-1.804529	-1.307213
C	-1.180509	-0.729481	-0.563496
C	-2.137440	0.024328	0.359131

N	-1.956725	1.395439	0.416385
C	-0.971743	2.169441	-0.210378
N	-0.127308	1.480211	-1.044482
C	-0.331233	0.135520	-1.541904
H	-2.557317	1.906970	1.055751
O	3.833682	0.758787	0.141445
O	0.011239	-0.418376	2.317620
H	2.260852	0.556279	2.026230
H	-0.380462	-2.971133	1.295088
H	1.298527	-3.019318	0.725000
H	-0.019678	-3.400386	-0.393976
O	-3.038037	-0.522308	0.969883
O	-0.876896	3.368012	0.004884
H	-1.362777	-2.363030	-2.014754
H	-2.435465	-2.504450	-0.600679
H	-2.794576	-1.332683	-1.873714
H	0.912809	-1.620658	-1.975088
H	-0.679325	0.145052	-2.583370
H	0.540798	2.076506	-1.518690
H	2.834322	-0.218975	-1.895547

30

Min S_0 CPD Neutral H_2O, E=-908.593981178 a.u., 0 imaginary frequencies

N	2.190027	-0.248819	-1.100774
C	2.693881	0.216264	0.068076
N	1.870930	0.043236	1.193165
C	0.648545	-0.588093	1.275218
C	0.157457	-1.302266	0.015112
C	0.212435	-2.808359	0.299861
C	0.863207	-0.787309	-1.268146
C	-2.051955	-1.711660	-1.337084
C	-1.199343	-0.687848	-0.575841
C	-2.098078	0.074631	0.389572
N	-1.864961	1.430261	0.490446
C	-0.901979	2.195960	-0.180422
N	-0.116060	1.523522	-1.059724
C	-0.339985	0.174952	-1.548326
H	-2.446697	1.948691	1.142229
O	3.797248	0.748515	0.187519
O	0.032282	-0.621844	2.333941
H	2.238502	0.437782	2.054408
H	-0.441439	-3.067418	1.137667
H	1.235229	-3.105995	0.557699
H	-0.092695	-3.384002	-0.577219
O	-2.998262	-0.454695	1.029103
O	-0.789899	3.398129	0.061778
H	-1.462797	-2.245775	-2.087997
H	-2.490864	-2.440839	-0.652411
H	-2.865437	-1.195745	-1.858208

H	0.901352	-1.565530	-2.032342
H	-0.689770	0.198623	-2.585185
H	0.526165	2.112263	-1.578684
H	2.792846	-0.147928	-1.908602

30

Min S_0 CPD Anion Gas Phase, E=-908.596525222 a.u., 0 imaginary frequencies

N	-1.919201	-0.394929	-1.401929
H	-2.182981	-0.217492	-2.362948
C	-2.651431	0.330235	-0.489174
O	-3.622726	1.024172	-0.812577
N	-2.221051	0.198558	0.813810
H	-2.802877	0.643986	1.512827
C	-1.357762	-0.834913	1.302752
O	-1.443531	-1.115895	2.514667
C	-0.534232	-1.460653	0.325498
C	-0.053932	-2.856207	0.606192
H	-0.635523	-3.606065	0.035924
H	1.001418	-3.018455	0.355910
H	-0.184168	-3.073627	1.670523
C	-0.579793	-0.918781	-1.097329
H	-0.404602	-1.751542	-1.793036
N	0.229820	1.455312	-0.851810
H	-0.598659	1.913849	-1.207585
C	0.783692	2.102773	0.221824
O	0.370609	3.195009	0.624727
N	1.860722	1.465065	0.796946
H	2.313062	1.943730	1.564330
C	2.481425	0.278235	0.335289
O	3.551427	-0.062772	0.885382
C	1.828327	-0.402777	-0.725130
C	2.542197	-1.523735	-1.412731
H	3.497605	-1.711423	-0.914093
H	2.743780	-1.276944	-2.472139
H	1.978276	-2.469435	-1.423807
C	0.556114	0.121945	-1.352258
H	0.688774	0.214532	-2.452213

30

Min S_0 CPD Anion H_2O, E=-908.683033822 a.u., 0 imaginary frequencies

N	1.871939	-0.572900	-1.312263
C	2.512672	-0.028958	-0.237226
N	1.859420	-0.202249	0.971684
C	0.849018	-1.181499	1.213740
C	0.145493	-1.629814	0.071738
C	-0.508826	-2.981703	0.095735
C	0.457825	-0.946827	-1.255048
C	-2.835576	-0.903298	-1.321375

C	-1.788543	-0.042673	-0.680186
C	-2.081084	0.648691	0.514154
N	-1.147916	1.639948	0.920260
C	-0.179810	2.196483	0.112258
N	0.028702	1.557866	-1.070993
C	-0.530993	0.265936	-1.469056
H	-1.386317	2.164241	1.753719
O	3.606088	0.555049	-0.313528
O	0.702842	-1.562976	2.403786
H	2.382578	0.094121	1.788092
H	-0.705539	-3.291823	1.126213
H	0.142345	-3.746894	-0.362444
H	-1.455621	-3.010790	-0.454752
O	-3.087335	0.479678	1.253345
O	0.438798	3.222932	0.442608
H	-2.397977	-1.683610	-1.954702
H	-3.471272	-1.384814	-0.572017
H	-3.496823	-0.308030	-1.974995
H	0.286856	-1.657515	-2.070245
H	-0.762484	0.347168	-2.540578
H	0.753793	1.965487	-1.647326
H	2.301249	-0.381674	-2.209862

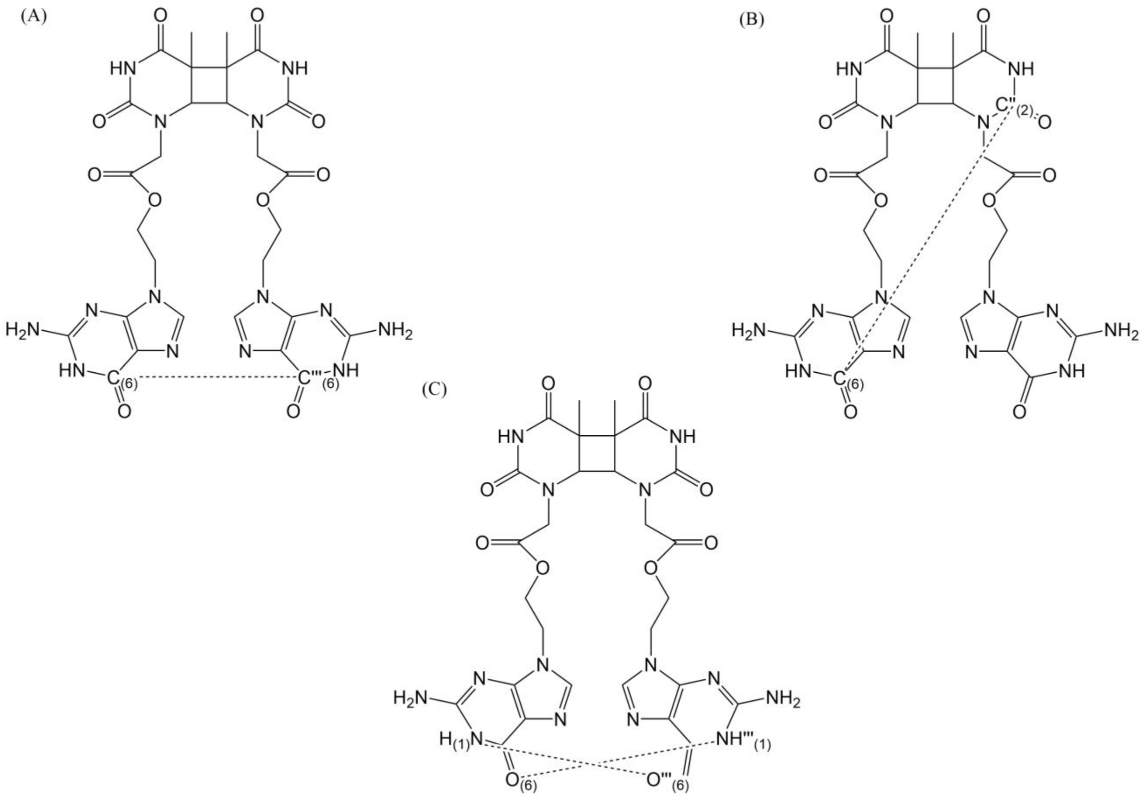

Figure S3. Atom numbering related to the G-CPD structures displayed in Figure 5.

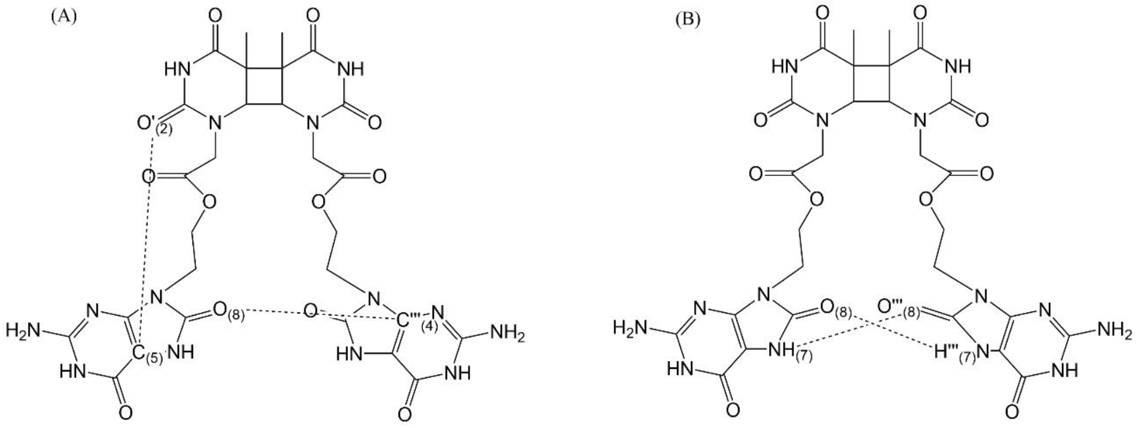

Figure S4. Atom numbering related to the OG-CPD structures displayed in Figure 6.
